# Beyond the tumor microenvironment: a hierarchical immune-circuit model of immune checkpoint blockade resistance

**DOI:** 10.3389/fonc.2026.1919294

**Published:** 2026-09-07

**Authors:** Xue Zhao, Han Wang, Yanan Liu, Lanqing Cao

**Affiliations:** 1 Department of Otorhinolaryngology, Head and Neck Surgery, The Second Hospital of Jilin University, Jilin University, Changchun, Jilin, China; 2 Department of Pathology, The Second Hospital of Jilin University, Jilin University, Changchun, Jilin, China

**Keywords:** biomarkers, dendritic cells, immune checkpoint blockade, immunotherapy resistance, microbiome, T-cell exhaustion, tumor microenvironment, tumor-draining lymph nodes

## Abstract

Immune checkpoint blockade (ICB) produces durable tumor control in a subset of patients, yet resistance is usually interpreted through tumor-intrinsic lesions or suppression within the tumor microenvironment (TME). We propose that ICB resistance can also be organized as failure of a multiscale antitumor immune circuit comprising local immune execution, regional immune education in tumor-draining lymph nodes (TDLNs), and systemic immune calibration by the host. In this model, “hierarchical” denotes nested functional dependence rather than one-way anatomical control: local killing depends probabilistically on antigen visibility, access, and a renewable supply of tumor-reactive cells; nodal priming depends on competent dendritic-cell licensing and host immune fitness; and reciprocal feedback can propagate or repair failure across compartments. We first define the canonical CD8-dominant substrate that ICB can amplify, including cross-presentation, costimulation, progenitor-exhausted T-cell maintenance, trafficking, and target-cell recognition, while retaining alternative CD4, natural killer, intratumoral antigen-presenting-cell, and tertiary lymphoid routes. We then evaluate local, regional, and systemic resistance mechanisms, four directional feedback axes, and context-dependent patterns in pancreatic cancer, melanoma, non-small-cell lung cancer, microsatellite-stable colorectal cancer, hepatocellular carcinoma, and prostate cancer. Evidence from animal perturbation, human spatial and clonal studies, and clinical trials supports individual circuit components but remains heterogeneous. Randomized perioperative regimens demonstrate disease- and setting-specific benefit, whereas negative randomized results and mixed or limiting early-phase signals across innate agonism, stromal or metabolic targeting, radiotherapy combinations, and systemic conditioning constrain therapeutic extrapolation. We therefore present compartment-resolved biomarkers and adaptive trial designs as research hypotheses, not a validated classifier or standard-care algorithm. The framework will be useful only if prespecified multiscale measurements improve prediction beyond tumor-only models and if mechanism-matched interventions produce the expected pharmacodynamic repair before clinical benefit is attributed to the circuit.

## 1 Introduction: resistance as failure of an immune circuit

Randomized trials show that immune checkpoint blockade (ICB)-containing regimens can improve outcomes in selected cancer settings (1, 2), yet target engagement at programmed cell death protein 1 (PD-1), programmed death-ligand 1 (PD-L1), or cytotoxic T-lymphocyte-associated protein 4 (CTLA-4) is not sufficient to ensure tumor regression. The antibodies release inhibitory constraints; they do not by themselves generate a recognizable antigen, a competent antigen-presenting cell, a renewable tumor-reactive lymphocyte population, a route into the tumor, or a target cell that remains susceptible to killing. Radiographic progression can therefore be the common endpoint of biologically different failures. Treating all such failures as additional suppression within the tumor microenvironment (TME) obscures where the response became unavailable and why the same combination may work in one setting but fail in another.

In the canonical, CD8-dominant response to PD-1-axis blockade, therapeutic expansion is disproportionately supported by progenitor-exhausted or stem-like CD8+ cells marked by T cell factor 1 (TCF1), whereas terminally exhausted populations have less proliferative and developmental capacity (3, 4). Human longitudinal studies further show that post-treatment tumor infiltrates can contain newly detected tumor-reactive clonotypes rather than simply expanded versions of every clone present in the pretreatment biopsy (5). These findings shift the explanatory question from whether a tumor contains any T cells to whether therapy can draw on a renewable, appropriately primed and trafficked cellular substrate. This route is canonical rather than exclusive: CD4+ T cells, natural killer cells, B-cell-rich tertiary lymphoid structures, and other effectors may compensate in particular tumors (6–8).

TME-centered models remain indispensable for explaining antigen-presentation loss, interferon-pathway lesions, immune exclusion, nutrient restriction, myeloid suppression, and terminal dysfunction. They are less complete, however, when resistance reflects failure to generate new clonotypes, loss of a progenitor reservoir in tumor-draining lymph nodes (TDLNs), impaired cell exchange between lymphoid tissue, blood, and tumor, or a host state that changes dendritic-cell and T-cell operating thresholds. Experimental lineage studies identify TDLNs as a source of stem-like tumor-specific CD8+ cells, while paired human studies support a two-stage program in which initial activation in lymph nodes can precede further differentiation in the tumor (9, 10). Intratumoral antigen-presenting-cell niches provide an important alternative maintenance site and prevent an exclusively nodal interpretation (7).

We therefore propose local execution in the tumor, regional education in TDLNs, and systemic calibration by the host as three compartments. “Hierarchical” denotes probabilistic nested dependency, not anatomical control: downstream killing may require upstream clone generation and physiological support, but reciprocal feedback and alternative routes add redundancy. Feedback is an interaction among the three compartments, not a fourth layer; several lesions can coexist and the candidate limiting factor can move during treatment.

Established cancer-immunity-cycle models map the sequential events required for antitumor T-cell immunity, while cancer-immune-set-point and immunogram frameworks integrate tumor, host, and environmental determinants (11–13). The present model does not replace them. Its proposed increment is source- and time-resolved causal localization: it asks where renewable clones are generated or maintained, distinguishes competing compartmental causes of the same tumor endpoint, and requires a compartment-matched intervention to repair its prespecified mediator before benefit is attributed to that compartment. It is therefore a falsifiable inference framework rather than another static checklist.

This testable hypothesis is not a staging system or clinical classifier. Its value depends on whether compartment-resolved measurements improve prediction or therapeutic selection beyond tumor-only models; negative outcomes cannot be rescued by retrospective relabeling as circuit failure.

## 2 Biological prerequisites for effective ICB

### 2.1 Antigen recognition, dendritic-cell licensing, and CD8+ T-cell priming

For a canonical CD8-dominant response, malignant cells must contribute antigens that can be captured, processed, and displayed by competent antigen-presenting cells. Conventional type 1 dendritic cells (cDC1) dependent on basic leucine zipper ATF-like transcription factor 3 (BATF3) are particularly efficient at cross-presenting cell-associated antigens and supporting cytotoxic T-cell priming; genetic or functional loss of this lineage impairs rejection of immunogenic tumors and checkpoint-based therapy in several mouse models (14, 15). Within tumors, cDC1 abundance may itself be limiting. After antigen acquisition, a migratory cDC1 population can upregulate C-C chemokine receptor type 7 (CCR7), enter afferent lymphatics, and deliver antigen to TDLNs, where it can present directly or transfer antigen to resident antigen-presenting cells (16). These perturbation studies define a plausible route, but their strongest causal evidence is preclinical; they do not establish universal cDC1 or TDLN dependence in patients.

The substrate begins even earlier, with tumor visibility. Antigen abundance is not equivalent to useful antigenicity: peptides must be processed and displayed on compatible human leukocyte antigen (HLA) molecules to be recognized by tumor-reactive T cells (17). Conversely, a productive response need not follow the identical CD8 route in every cancer, because cross-presented antigen can coexist with direct presentation, CD4+ help, natural-killer-cell activity, or local lymphoid organization (6–8). This redundancy is biologically protective but complicates biomarker interpretation; no single antigen or dendritic-cell measurement establishes completion of the priming sequence.

Antigen display alone is insufficient. Productive priming couples peptide–major histocompatibility complex (MHC) recognition to CD80/CD86–CD28 costimulation and cytokine licensing in the appropriate spatial and temporal window. Conditional experiments show that proliferative rescue by PD-1 blockade can remain CD28 dependent, with supportive pharmacodynamic observations in patients with non-small-cell lung cancer (18). Type I interferon signaling directly in cross-presenting dendritic cells can support tumor-specific priming (19), while anti-PD-1 activity in defined mouse tumors requires reciprocal communication in which T-cell-derived interferon-γ (IFN-γ) induces dendritic-cell interleukin-12 (IL-12), and IL-12 sustains effector function (20). Thus, “inflammatory context” should mean functional maturation, costimulation, and a specified cytokine source—not inflammation in the abstract. These programs are neither monotonic nor universal: persistent interferon can install secondary inhibitory states, and innate activation can induce PD-L1 alongside costimulatory molecules.

### 2.2 A renewable ICB-responsive T-cell substrate

Chronic antigen exposure produces heterogeneous dysfunctional CD8+ states. TCF1-positive progenitor-exhausted cells retain self-renewal, proliferative competence, and the capacity to generate more differentiated progeny, whereas terminally exhausted cells are less responsive to checkpoint release (3, 4). ICB should therefore be understood less as wholesale reversal of a fixed exhaustion program than as selective expansion and differentiation of cells that remain developmentally competent. Consistent with this view, post-treatment exhausted populations in human skin cancers often included clonotypes not detected in the matched pretreatment sample (5). Sampling limits preclude assigning every new clone to an extracancer source, but the observation makes continued recruitment biologically relevant.

The renewable pool is anatomically distributed. TDLNs can contain stable TCF1-positive tumor-specific reservoirs related to differentiated intratumoral progeny (9). Intratumoral antigen-presenting-cell niches can maintain stem-like states, whereas tertiary lymphoid structures are associated with memory-like T-cell organization and favorable ICB outcomes (7, 8). The relevant question is therefore not whether the node or tumor is the sole reservoir, but which site preserves or marks functional clones in a particular disease state.

### 2.3 Trafficking and metabolic support complete the response cycle

Primed cells must leave lymphoid tissue, circulate, extravasate, penetrate tumor parenchyma, recognize malignant targets, and retain sufficient bioenergetic capacity to kill. Human blood–tumor clonal overlap supports peripheral replenishment, whereas *in vivo* labeling demonstrates continuous, bidirectional movement of TCF1-positive cells between tumor and lymphoid tissue in mouse models (21, 22). A lesion at any step can therefore yield progression despite pharmacological checkpoint engagement. Serial T-cell receptor (TCR) tracking, spatial localization, antigen-presentation assays, and cell-specific metabolic readouts are more informative for locating failure than a single pretreatment count of total CD8+ cells.

## 3 Definition and testable principles of the multiscale immune circuit

### 3.1 Three compartments, one nested dependency

**Figure 1:**
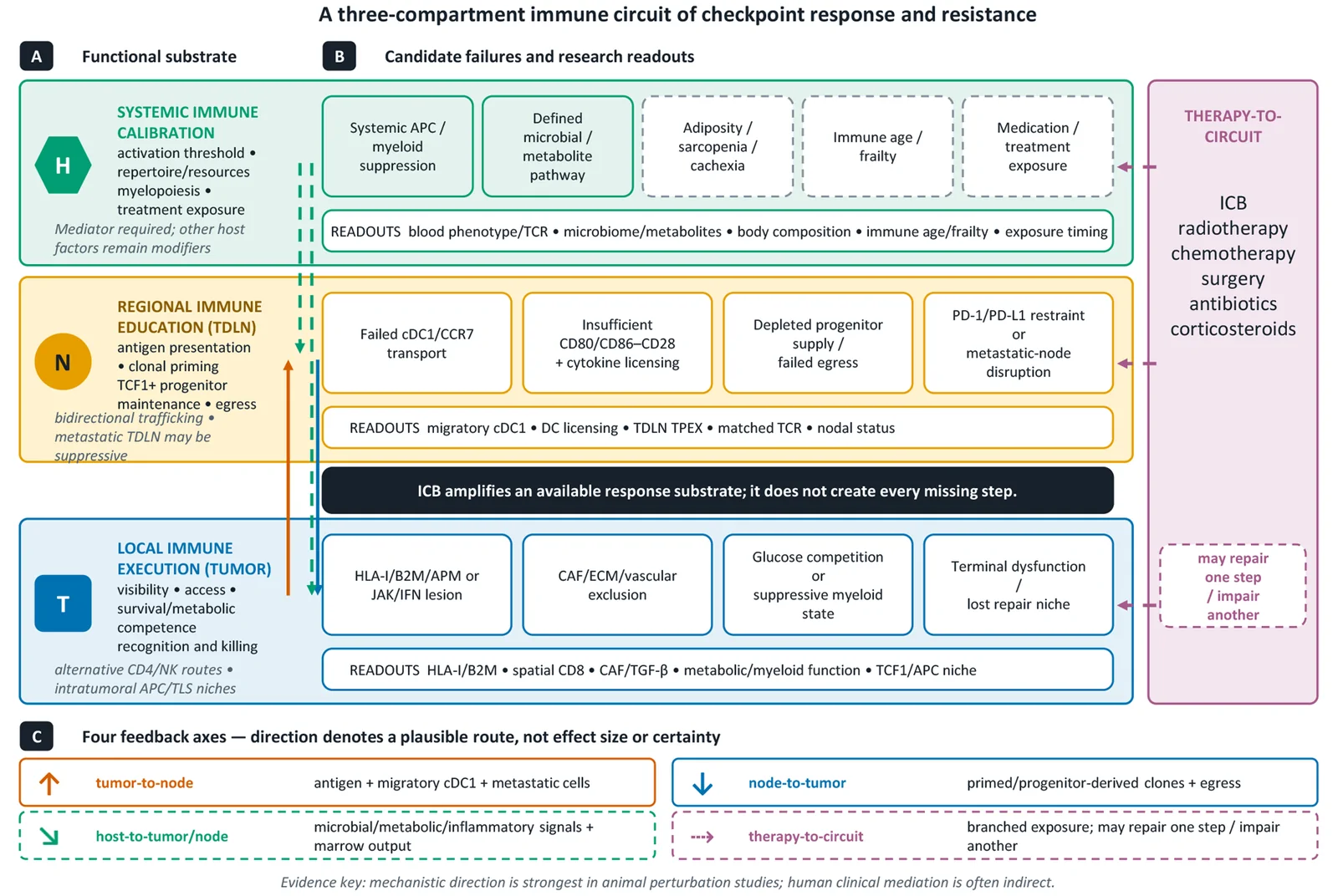
A three-compartment immune circuit of checkpoint response and resistance. **(A)** Functional substrate across systemic, regional, and local compartments. **(B)** Candidate failures and research readouts. **(C)** Four directional feedback axes. The model separates local immune execution in the tumor bed, regional immune education in tumor-draining lymph nodes (TDLNs), and systemic immune calibration by host-wide immune, metabolic, microbial, and treatment-related states. In the canonical CD8-dominant route, tumor antigen is captured by competent antigen-presenting cells; CCR7-dependent cDC1 migration and appropriate costimulation/cytokine licensing support clonal priming; TCF1-positive progenitor states generate differentiated progeny that traffic to tumors; and local access, metabolic competence, target recognition, and cytotoxic function permit immune checkpoint blockade (ICB) to amplify an available response substrate (3, 9, 16, 20). The four labeled feedback axes are: tumor-to-node, through antigen/migratory-DC transport and metastatic-cell entry; node-to-tumor, through clonal expansion, progenitor differentiation, and egress; host-to-tumor/node, through microbial, metabolic, inflammatory, repertoire, and myeloid signals; and therapy-to-circuit, through interventions that may repair one step while impairing another (24–28). Major candidate failures include loss of local visibility, access, metabolic/myeloid competence, or renewable T-cell states; defective regional antigen transport/licensing, progenitor supply/egress, or nodal organization; and mediator-linked systemic APC, myeloid, or microbial pathways. Body composition, age/frailty, and treatment exposures remain contextual, prognostic, or safety modifiers unless a mediator to the ICB-responsive substrate is demonstrated. The arrows denote plausible, context-dependent biological routes, not universal requirements or a validated treatment sequence. Intratumoral APC/TLS niches, alternative CD4/NK effectors, bidirectional trafficking, and suppressive metastatic nodes provide explicit boundary conditions (6–8, 22).

The model separates three connected compartments. Local immune execution covers antigen recognition, tissue access, survival, and killing in the tumor bed; regional immune education covers tumor-antigen transport, priming, progenitor maintenance, and egress in TDLNs; systemic immune calibration covers host states that affect activation thresholds, repertoire, myelopoiesis, metabolism, and trafficking. They are not ranked anatomically. Hierarchy means nested probabilistic dependence: a permissive tumor may remain undersupplied when regional priming fails, whereas nodal clones may be ineffectual when tumor entry or recognition is lost. Intratumoral niches, bidirectional trafficking, and disseminated immunity add redundancy (7, 22, 23). **Figure 1** summarizes the compartments and four feedback axes; **Table 1** defines their mechanisms, candidate measurements, and evidentiary limits.

**Table 1:** Operational definitions, failure modes, and candidate readouts across the three immune-circuit compartments.

Compartment and operational definition	Principal failure mechanisms	Candidate diagnostic or pharmacodynamic readouts	Evidence level, directness, and boundary
Local immune execution Recognition, access, survival, and killing within the tumor bed	• Loss of tumor visibility: HLA-I/B2M/APM or IFN-γ/JAK defects • Stromal/vascular exclusion and chemokine mismatch • Nutrient competition and suppressive myeloid states • Terminal dysfunction or loss of supportive APC/TLS niches	• HLA-I/B2M/APM integrity; paired JAK/STAT/IFN-response testing • Stromal-versus-parenchymal CD8 localization; CAF/TGF-β/ECM and perfusion state • Tumor/T-cell glucose use; myeloid PI3Kγ target engagement and function • TCF1-positive progenitor-to-terminal-state ratio; APC-niche/TLS organization (3, 4, 7, 8)	• Paired human resistance and spatial evidence: B–C; causal mechanisms often D (17, 29–32) • B2M loss is not deterministic when CD4/NK routes compensate (6) • An invasive-margin CD8 signal may indicate responsive pre-existing immunity, not exclusion (33) • Pathway plausibility does not establish drug efficacy; ECHO-301 was negative (34)
Regional immune education Antigen transport, clonal priming, differentiation, progenitor maintenance, and egress in tumor-draining lymph nodes (TDLNs)	• Defective cDC1 antigen capture, CCR7-dependent transport, or antigen handoff • Inadequate CD80/CD86–CD28 costimulation or cytokine licensing (18–20) • Loss of TCF1-positive progenitor supply or impaired egress • PD-1/PD-L1 restraint, suppressive remodeling, or metastatic-node disruption (25, 35)	• Shared tumor–node antigen; migratory cDC1 location and CCR7 trajectory • DC CD80/CD86, IL-12 source, type I IFN program, and DC–T-cell contact • TDLN TPEX state; matched node–blood–tumor TCR clonotypes and temporal transitions • Nodal involvement, DC/TPEX spatial organization, suppressive niches, and circulating intermediates	• Causal transport, lineage, resection, and egress evidence is mainly D (9, 16, 24) • Human paired/spatial evidence is B–C and does not randomize nodal preservation (10, 25) • TDLNs are not uniformly productive; metastatic nodes can be suppressive, and intratumoral APC/TLS niches provide anatomical redundancy (7, 8) • No practice-changing clinical evidence was identified in this targeted synthesis; indicated nodal surgery and radiotherapy take precedence
Systemic immune calibration Host states with defined mediators that alter activation thresholds, myelopoiesis, repertoire, metabolism, or trafficking across tumor and TDLN	• Causal candidates: systemic APC hyporesponsiveness or suppressive myelopoiesis; defined microbiome/metabolite pathways • Unvalidated correlates: adiposity, sarcopenia/cachexia, and chronological or immune age • Context/confounding: antibiotics, corticosteroids, chemotherapy, radiotherapy, and surgery • Safety/prognosis: frailty and treatment tolerance, not presumed immune lesions	• Blood immune phenotype, TCR clonality/diversity, proliferative response, and APC/myeloid function • Standardized microbiome/metabolite measures with diet, drug, geography, and batch covariates • CT/DXA body composition, weight trajectory, inflammation, and cachexia • Naive-cell supply, immune-age measures, frailty, and treatment-exposure timing	• Organism-wide coordination and microbiome causality are strongest in mice (D); human signatures are mainly C (28, 36–39) • FMT studies provide early uncontrolled intervention evidence (B), not established added efficacy (40–42) • BMI, age, frailty, one taxon, or one metabolite is not a resistance classifier (43–45) • Medication associations are vulnerable to timing, indication, and disease-severity confounding (46–48) • Only a mediator-linked host state is a candidate circuit lesion; other factors remain covariates or safety modifiers

These compartments are analytically distinct but biologically coupled. “Hierarchical” denotes nested functional dependency with context-dependent redundancy, not a one-way anatomical command chain. Candidate readouts are research variables unless independently validated for a defined assay, disease, setting, and treatment. APC, antigen-presenting cell; APM, antigen-processing machinery; B2M, β2-microglobulin; BMI, body mass index; CAF, cancer-associated fibroblast; CD4, cluster of differentiation 4; CD8, cluster of differentiation 8; CD28, cluster of differentiation 28; CD80, cluster of differentiation 80; CD86, cluster of differentiation 86; cDC1, conventional type 1 dendritic cell; CCR7, C-C chemokine receptor type 7; CT, computed tomography; DC, dendritic cell; DXA, dual-energy X-ray absorptiometry; ECM, extracellular matrix; FMT, fecal microbiota transplantation; HLA-I, human leukocyte antigen class I; IFN, interferon; IL, interleukin; JAK, Janus kinase; NK, natural killer; PD-1, programmed cell death protein 1; PD-L1, programmed death-ligand 1; PI3Kγ, phosphoinositide 3-kinase-γ; STAT, signal transducer and activator of transcription; TCF1, T cell factor 1; TCR, T-cell receptor; TDLN, tumor-draining lymph node; TGF-β, transforming growth factor-β; TLS, tertiary lymphoid structure; TPEX, progenitor-exhausted T cell.

Operationally, the hierarchy is defined by conditional failure patterns. Preserved tumor access and visibility with absent expansion of tumor-matched nodal progenitors and no subsequent blood emergence nominates regional supply failure. Nodal and blood expansion without tumor entry instead nominates trafficking or local access failure; tumor arrival without recognition or killing nominates local execution failure. Expansion from intratumoral niches or non-draining lymphoid sites despite absent TDLN output would weaken strict nodal dependence. These observations localize a conditional dependency; anatomy alone does not establish dominance.

### 3.2 Four testable principles

The necessary-substrate principle states that ICB can amplify only an available response substrate. Within the canonical CD8-dominant route, deficiencies in antigen presentation, dendritic-cell licensing, clone generation, trafficking, or metabolic competence should reduce response probability, while alternative effector routes may compensate. The candidate-limiting-factor principle predicts that one impaired step may disproportionately constrain the circuit in a defined context, without invoking a literal minimum law: several submaximal lesions may combine, and the dominant constraint may move after treatment. The feedback principle predicts that outputs of one compartment reshape the others—for example, nodal clones seed tumors and tumor-derived material conditions nodes—while explicitly retaining only three failure compartments. The context principle predicts that tumor type, genotype, disease stage, metastatic site, prior therapy, and host state reorder candidate limitations.

These propositions are useful only if falsifiable. A compartment-resolved model should outperform tumor-only measures; nodal supply requires progenitor expansion before matched clones appear in blood and tumor; and mechanism attribution requires the prespecified pharmacodynamic repair. Evidence supports individual components, but our targeted search through 15 August 2026 identified no validation of the complete hierarchy as a classifier. The framework should guide measurement and trial design, not fixed layer assignment.

Because sparse intratumoral effectors can reflect failed priming, egress, access, recognition, or survival, causal localization requires temporal ordering plus a perturbation or natural experiment. Cross-sectional correlation can nominate a route but cannot identify where failure began.

## 4 Local execution failure: when effectors cannot recognize, enter, or kill

### 4.1 Tumor visibility and interferon responsiveness

Local execution first requires that malignant cells remain visible and responsive to immune pressure. Paired melanoma samples obtained before pembrolizumab and at relapse identified acquired Janus kinase 1/2 (JAK1/JAK2) lesions that impaired interferon-γ (IFN-γ) responsiveness and β2-microglobulin (B2M) loss that disrupted surface major histocompatibility complex class I (MHC-I) presentation (17). Such lesions can disconnect successful priming and trafficking from target-cell recognition or susceptibility, thereby producing acquired resistance downstream of an otherwise active circuit. Their prevalence and determinism should not be overstated: the report comprised four patients, and selected B2M-deficient models retained sensitivity through compensatory CD4+ T-cell or natural-killer-cell routes (6). Assessment should therefore combine paired sequencing with surface human leukocyte antigen class I (HLA-I)/B2M, functional IFN-γ-response assays, and the presence of alternative effectors rather than treating one genotype as a universal endpoint.

Interferon biology also depends on duration and cellular compartment. Acute type I interferon signaling in dendritic cells can license cross-presentation, and T-cell IFN-γ can promote interleukin-12 (IL-12)-producing dendritic-cell states that support anti-PD-1 activity (19, 20). By contrast, sustained type I or type II interferon signaling can induce multigenic inhibitory programs, inducible nitric oxide synthase (NOS2)-associated suppression, and secondary resistance in experimental systems with human biomarker concordance (49, 50). Neither global interferon stimulation nor blockade follows from these observations. Serial, cell-resolved interferon-stimulated-gene measurements are needed to distinguish early licensing from chronic adaptive resistance.

### 4.2 Stromal and vascular access

Even competent effectors cannot execute if tissue architecture prevents contact with malignant cells. Live imaging of human lung-tumor slices showed that dense, aligned collagen and fibronectin could retain T cells in stromal regions and restrict movement into epithelial tumor islets (29). In metastatic urothelial cancer, fibroblast transforming growth factor-β (TGF-β) signaling was associated with CD8+ retention in collagen-rich peritumoral stroma and nonresponse to atezolizumab; pathway blockade restored central penetration and cooperated with PD-L1 blockade in a mouse model (30). These findings support a spatial access bottleneck only when stromal retention and deficient parenchymal or tumor-islet entry are demonstrated.

Margin localization alone is not evidence of failed access. In metastatic melanoma, a pretreatment CD8+ population at the invasive margin together with intratumoral cells marked pre-existing adaptive immunity and was associated with pembrolizumab response (33). Moreover, indiscriminate depletion of stromal or fibroblast compartments accelerated pancreatic cancer in experimental models, showing that stroma can restrain as well as support disease (51, 52). Spatial CD8 distribution, extracellular-matrix architecture, fibroblast state, TGF-β activity, perfusion, and endothelial access should therefore be measured together before a tumor is labeled “excluded” or assigned a remodeling strategy.

Access is a sequence rather than a scalar infiltration score: cells must be delivered by a perfused vessel, cross an activated endothelium, traverse interstitial matrix, and enter malignant-cell-rich regions (29, 30). A high total lymphocyte density can conceal failure at the final step, whereas a low-density biopsy can reflect sampling of a heterogeneous lesion. Paired spatial maps before and during therapy should therefore preserve stromal, vascular, and tumor-islet coordinates and test whether an intervention changes physical localization, not merely bulk expression of an inflammatory signature.

### 4.3 Metabolic and myeloid paralysis

Local suppression can persist after effectors reach the tumor. In a mouse sarcoma model, tumor-cell glucose consumption lowered glucose availability, T-cell mechanistic target of rapamycin (mTOR) activity, glycolysis, and IFN-γ production; checkpoint blockade altered this competition and restored T-cell metabolic function (31). This establishes a causal glucose-competition mechanism in the tested system, not a universal inference from blood glucose. A translational test would require compartment-specific glucose uptake and availability together with T-cell glycolytic and effector readouts.

A second bounded example is suppressive myeloid phosphoinositide 3-kinase-γ (PI3Kγ) signaling. Genetic and pharmacological perturbation in mouse tumors reprogrammed myeloid cells, increased cytotoxic T-cell activity, and restored checkpoint sensitivity (32). Yet mechanistic plausibility does not guarantee a successful inhibitor regimen. In the phase III ECHO-301/KEYNOTE-252 trial, adding the indoleamine 2,3-dioxygenase 1 (IDO1) inhibitor epacadostat to pembrolizumab improved neither progression-free nor overall survival in advanced melanoma (34). That result directly refutes the tested combination, dose, and setting, while neither disproving all tryptophan–kynurenine biology nor validating glucose- or PI3Kγ-directed treatment in humans. Broader metabolic and myeloid candidates should remain in comparative tables until each is linked to target engagement, a functional cellular change, and clinical outcome.

### 4.4 Exhaustion and intratumoral repair niches

Persistent antigen and suppressive signals can drive infiltrating CD8+ cells toward terminal dysfunction, but exhaustion is not a single irreversible state. TCF1-positive progenitor-exhausted cells self-renew and generate more differentiated progeny during vaccination or checkpoint therapy, whereas terminally exhausted cells contribute less to durable expansion (3, 4). Total CD8+ density can therefore conceal the absence of a renewable substrate. State-resolved phenotyping, clonal continuity, and early proliferative response should accompany simple cell counts.

Renewal can also be supported locally. Human tumors contain antigen-presenting-cell-rich niches that maintain stem-like CD8+ cells, and B-cell-rich tertiary lymphoid structures associate with memory-like T-cell organization and favorable immunotherapy outcomes (7, 8). These observations constrain any claim that responsive cells must originate in TDLNs, although association cannot establish local clonal origin. Local remodeling is most likely to succeed when an expandable pool and viable trafficking route remain; matched tumor–node TCR lineages and spatial niche mapping could estimate their relative contributions.

## 5 Regional education failure: antigen transport, clone supply, and TDLN corruption

### 5.1 Tumor-to-node antigen and dendritic-cell transport

TDLNs connect local antigen release to regional T-cell education. In mouse melanoma, cDC1 that acquire tumor antigen adopt CCR7-dependent migratory programs, enter afferent lymphatics, and reach draining nodes, where they present antigen directly and transfer it to resident antigen-presenting cells. Conditional loss of CCR7 in this lineage impairs nodal priming and tumor control (16). Expansion and scheduled activation of tumor cDC1 can likewise increase antigen transport, tumor-specific CD8+ priming, and sensitivity to PD-L1-based treatment in the tested models (53). These studies provide a mechanistic bridge from the tumor to the node, but neither CCR7 expression nor cDC1 abundance alone proves that antigen arrived, was productively displayed, or generated a tumor-reactive clone in a patient.

Migration is also not the only productive fate of an activated dendritic cell. Photoconversion and spatial studies identify CCR7-positive populations that remain within tumors, interact with CD8+ cells, and occupy locally stimulatory or reversibly suppressed niches (54, 55). A useful assay must therefore distinguish marker expression from trajectory by measuring tumor-to-node antigen sharing, dendritic-cell location, and direct versus handed-off presentation. Regional education is major but nonexclusive: antigen presentation and T-cell support can occur in the node, tumor, and tertiary lymphoid structures (7, 8).

Once in the node, antigen display must be coupled to functional licensing. B7–CD28 costimulation remains necessary for proliferative rescue by PD-1 blockade in relevant models, while T-cell IFN-γ and dendritic-cell IL-12 can form a reciprocal circuit that sustains antitumor activity (18, 20). Type I interferon can act directly on cross-presenting dendritic cells to support priming (19). However, innate activation can simultaneously induce stimulatory CD86 and inhibitory PD-L1, so nodal cytokine abundance cannot substitute for cell-specific measurements of CD80/CD86, IL-12 source, PD-L1, and dendritic-cell–T-cell contact (56).

### 5.2 Clonal priming, progenitor maintenance, and node-to-tumor egress

TDLNs can serve as sites of initial activation and long-term clone supply rather than passive conduits. In mouse lung tumors, stable TCF1-positive tumor-specific nodal populations were clonally and developmentally upstream of more differentiated intratumoral cells (9). Studies integrating human kidney and prostate cancers with mouse perturbation similarly support an initial stem-like activation phase in lymph nodes followed by additional costimulation and effector differentiation within the tumor (10). This two-stage route explains how a locally accessible tumor may remain under-supplied, but it is not universal: intratumoral antigen-presenting-cell niches and tertiary lymphoid structures can maintain responsive states (7, 8). Matched node–blood–tumor TCR tracking is needed to determine which reservoir contributes in a given context.

Supply also requires egress. In selected mouse models, removing the TDLN or using the sphingosine-1-phosphate receptor modulator FTY720 to sequester lymphocytes abolished PD-1/PD-L1-mediated tumor regression and reduced tumor infiltration (24). Human multicompartment data show that peripheral expansion of shared effector clonotypes accompanies tumor infiltration and clinical response, consistent with recruitment from outside the sampled tumor (21). FTY720, however, affects multiple lymphocyte subsets and trafficking routes and cannot be interpreted as a selective clinical test of TDLN export. Moreover, *in vivo* labeling shows continuous traffic in both directions between tumors and lymphoid tissues (22), and the original TDLN can become dispensable after antigen-specific cells have disseminated to other lymphoid organs in specialized mouse settings (23). The measurable prediction is temporal: expansion of tumor-matched nodal progenitors should precede their detection in blood and subsequent intratumoral effector accumulation when regional supply is operative.

### 5.3 Conversion of a priming site into a tolerogenic or metastatic niche

A draining node can be productive and checkpoint restrained at the same time. In mouse models, tumor-specific PD-1-positive T cells interact with PD-L1-positive nodal conventional dendritic cells, and TDLN-directed PD-L1 blockade increases progenitor-exhausted cells that subsequently seed tumors; corresponding cellular contacts in nonmetastatic melanoma nodes were associated with recurrence (35). This chain places the PD-1/PD-L1 interaction within the regional compartment without making every TDLN suppressive. Nodal PD-1/PD-L1 contact, dendritic-cell costimulation, progenitor expansion, and outbound tumor-matched clonotypes should be measured together to distinguish antigen engagement from functional paralysis.

Metastatic colonization can qualitatively change that balance. A prospective neoadjuvant anti-PD-L1 study in head and neck cancer found abundant, dendritic-cell-adjacent TCF1-positive progenitor-exhausted cells and treatment-associated intermediate states in uninvolved regional nodes, whereas metastatic nodes showed disrupted responses and suppressive spatial niches (25). In resectable lung cancer, tumor and nodal responses could diverge, and involved nodes were associated with immune exclusion, poorer primary-tumor pathological response, and relapse (57). These observational data do not show that preserving or removing a particular node causes clinical benefit. They instead require distinction among uninvolved, tumor-conditioned, microscopically involved, and overtly replaced nodes; anatomical presence is not a proxy for immune competence.

### 5.4 Repairing or preserving regional immunity under therapy

Regional repair strategies should be evaluated as explicit causal cascades. Tumor antigen release or delivery must be followed by innate sensing, type I interferon signaling, dendritic-cell maturation, cross-presentation and costimulation, clonal expansion, egress, and tumor re-entry. In immunogenic mouse tumors, tumor-derived DNA activates host cyclic GMP–AMP synthase (cGAS)–stimulator of interferon genes (STING)–interferon regulatory factor 3 (IRF3) signaling, and type I interferon acting on dendritic cells supports cross-priming (19, 58). Toll-like receptor (TLR) agonism can converge on dendritic-cell maturation, while FMS-like tyrosine kinase 3 ligand expansion or CD40 activation can increase the available antigen-presenting-cell substrate (15, 59). Myeloid or macrophage reprogramming is a distinct, indirect route: phosphoinositide 3-kinase-γ inhibition shifted suppressive myeloid programs and restored T-cell activity in mouse tumor models (32), but this evidence does not establish recovery of TDLN dendritic-cell cross-priming or human efficacy. These approaches are not interchangeable, and TLR stimulation can coinduce PD-L1 (56). Each proposed *in situ* vaccination or node-directed intervention therefore requires evidence for every transition rather than assuming that innate activation culminates in useful ICB-responsive clones.

Radiotherapy belongs at this tumor-to-node and therapy-to-circuit interface. Tumor-directed radiation can release antigen and cytosolic DNA, activate cGAS–STING, induce type I interferon in dendritic cells, and support cross-presentation in mouse models (26). The effect is nonmonotonic: sufficiently large fractions can induce three-prime repair exonuclease 1 (TREX1) and attenuate cytosolic-DNA sensing (60). Field also matters. Concurrent elective irradiation of draining nodes reduced immune infiltration and the efficacy of combined radiation and checkpoint blockade in preclinical systems (27). Translation remains unsettled because randomized phase II trials of stereotactic radiation plus PD-1 blockade have produced mixed or negative results, including no improvement in nonirradiated-lesion response, progression-free survival, or overall survival in an unselected head and neck cancer population (61, 62). Dose, fractionation, target, nodal exposure, and disease context must therefore be prespecified.

Locoregional delivery of checkpoint antibodies to lymph nodes improved nodal and tumor pharmacodynamics at lower systemic exposure in mouse models, providing proof of regional therapeutic addressability rather than human efficacy (63). Similarly, preclinical studies showing that elective nodal irradiation can impair antitumor immunity motivate lymphatic-aware sequencing and radiation research (27, 64), but metastatic nodes may be dysfunctional and regional control remains an oncological priority (25). These findings do not justify omitting indicated lymphadenectomy or altering standard nodal radiation fields. Prospective studies should instead relate incidental uninvolved-node dose or locoregional drug exposure to nodal dendritic-cell/progenitor-exhausted T-cell states, systemic clone emergence, regional control, and survival. TDLNs are major but nonexclusive immune organs, and preserving anatomy is meaningful only when function and cancer safety are preserved together.

## 6 Systemic calibration failure: host state changes the operating threshold

Systemic calibration is restricted here to host states with a defined mediator linking an exposure or physiological state to the ICB-responsive substrate. Organism-wide perturbation studies show that antitumor therapy can require coordinated peripheral immunity and that tumors can impose reversible antigen-presenting-cell dysfunction through interleukin-1 (IL-1)- and granulocyte colony-stimulating factor (G-CSF)-linked programs (28, 36). Human blood studies detect early T-cell reinvigoration and recruitment of clonotypes during PD-1 blockade, but a circulating signal can reflect nodal export, tumor release, marrow output, redistribution, or toxicity rather than mediate regression (5, 65). A systemic factor is therefore a candidate circuit lesion only when a target cell or mediator, a predicted regional or local consequence, and a discriminating pharmacodynamic readout are specified.

The retained examples occupy different inferential categories. Microbial and myelopoietic pathways are causal candidates when perturbation identifies a dendritic-cell, myeloid, or T-cell mediator; body composition and chronological age are generally unvalidated correlates unless linked to a cellular pathway; frailty and treatment tolerance are prognostic or safety modifiers; and antibiotic or corticosteroid exposure is strongly vulnerable to indication bias. Treatment tolerance is therefore analyzed separately from biological ICB resistance. Each subsection asks whether the chain from exposure or state to mediator, downstream compartment, measurement, and alternative explanation can be completed.

### 6.1 Microbial signals as candidate antigen-presenting-cell and lymphocyte calibrators

The microbiome illustrates why evidence levels must remain separate. Responder-to-mouse fecal transfer and bacterial rescue experiments support causal modulation of PD-1 activity in mice, including an IL-12-associated immune route, whereas cross-cohort human studies find ecosystem-level associations that vary with geography and cohort composition (37, 38). Diet, medication exposure, sample handling, sequencing methods, and tumor type can all alter the measured community. A reproducible “responder microbiome” has therefore not emerged, and a single taxon should not be used as an ICB eligibility marker. Prospective studies should predefine community-level features, record diet and relevant drugs, standardize collection and analysis, and require external validation before assigning predictive meaning.

Human intervention establishes manipulability more clearly than efficacy. In small, uncontrolled melanoma studies, fecal microbiota transplantation (FMT) with anti-PD-1 therapy produced engraftment, immune changes, and responses in some patients (40, 41). These signals coexist with a small phase IIa cohort that reported no objective responses (42). Donor selection, treatment line, recipient engraftment, concurrent ICB, and the absence of an ICB-only control limit attribution. Microbial metabolites can also reverse direction across compartment and checkpoint context: systemic short-chain fatty acids were associated with impaired CTLA-4 blockade in one mixed mouse-human study, whereas fecal short-chain fatty acids correlated with favorable PD-1 outcomes in another (66, 67). No included study directly closes a human commensal or metabolite to tumor-reactive TCF1-positive reservoir-maintenance chain. Trials should therefore measure engraftment and a prespecified dendritic-cell or T-cell mediator and avoid inferring that microbial change restored regional clone supply.

### 6.2 Metabolic–inflammatory calibration versus body-composition proxies

Body size is similarly an imprecise proxy for the immune and physiological states relevant to ICB. Obesity increased leptin-associated T-cell dysfunction in experimental systems yet was associated with favorable PD-1 outcomes in selected retrospective cohorts, creating an apparent obesity paradox (68, 69). A large multicenter melanoma cohort did not reproduce an obesity benefit and instead associated muscle quality, underweight status, and visceral adiposity with outcome (43). Sarcopenia has also marked poor outcomes in prospective but small non-small-cell lung cancer (NSCLC) cohorts, although aggressive disease can itself cause muscle loss and inflammation (70). These data justify measuring visceral fat, muscle density, weight trajectory, cachexia, inflammatory state, sex, and tumor burden. They do not justify classifying resistance or benefit from body mass index (BMI) alone or assuming that body-composition intervention will restore ICB response.

### 6.3 Repertoire and cellular-supply constraints with aging

Aging can alter cellular supply without making chronological age a resistance category. In aged mice, impaired dendritic-cell migration and reduced naive CD8+ T-cell availability accompanied failure of PD-1/CTLA-4 therapy, while experimental dendritic-cell hyperactivation restored tumor control through a predominantly cytolytic T helper 1 (TH1)-polarized CD4+ T-cell route (44). The model-specific rescue is direct mechanistic evidence, but it is not proof of a universal human CD8+ T-cell defect. In a 50-patient melanoma cohort, older and younger responders showed different early TCR-repertoire dynamics, without evidence that either pattern caused response (45). Frailty may identify hospitalization and mortality risk more effectively than age alone, yet it primarily reflects reserve and safety and is not a validated efficacy mechanism (71). Immune-age studies should measure dendritic-cell migration, naive repertoire, effector expansion, frailty, and treatment tolerance as distinct variables.

### 6.4 Treatment-induced perturbation, confounding, and tolerance

Therapies and supportive medications can recalibrate the same circuit that ICB is intended to activate. Prior antibiotics and baseline corticosteroids have correlated with inferior ICB outcomes, but indication, timing, infection, palliative burden, and disease severity materially confound these associations (46, 47). In NSCLC, the adverse steroid association was concentrated among patients treated for cancer-related palliative indications, weakening a simple causal interpretation (48). Chemotherapy and surgery can alter microbiota or systemic inflammatory programs in causal mouse experiments, but these effects vary by agent and model and do not explain perioperative efficacy by themselves (72, 73). Exposure-aware trials should record indication, dose, timing, longitudinal immune and microbial changes, and treatment-specific outcomes. Analyses should treat exposure as time varying and separate response efficacy from infection, toxicity, hospitalization, and treatment-tolerance endpoints. These observations do not support withholding medically indicated antibiotics, corticosteroids, surgery, chemotherapy, or radiotherapy.

## 7 Four cross-compartment feedback axes

Feedback is used here to mean directional influence, not mere covariation. The four axes and their directions are summarized in **Figure 1**. Each axis should specify a source, a mediator or cellular carrier, an expected temporal order, a boundary condition, and a measurable consequence. This constraint prevents the model from gaining explanatory flexibility simply by adding arrows: if the proposed upstream change does not precede its downstream readout, that feedback mechanism is weakened in the tested context.

Tumor-to-node feedback begins when tumor-derived material reaches regional lymphoid tissue. CCR7-dependent migratory dendritic cells can transport tumor antigen to draining nodes in mouse models, whereas overt nodal metastasis in human head and neck cancer can disrupt the spatial relationship between dendritic cells and TCF1-positive progenitor-exhausted cells (16, 25). Antigen delivery and nodal corruption can thus operate in the same anatomical basin, so node involvement cannot be interpreted as either productive or suppressive from anatomy alone. Premetastatic conditioning, microscopic involvement, and overt replacement should be analyzed as distinct biological states under the umbrella label “node positive.” Human cross-sectional observations locate these states but cannot by themselves establish whether altered nodal organization preceded progression or resulted from it. Serial measurement of shared antigen, nodal involvement, dendritic-cell position, progenitor state, and treatment timing would test whether tumor evolution changes regional education before altered ICB response.

Node-to-tumor feedback concerns the supply of newly activated or maintained clones. Lineage studies place stable nodal TCF1-positive tumor-specific populations upstream of more differentiated intratumoral progeny, and perturbing the draining node or lymphocyte egress can diminish checkpoint activity in selected models (9, 24). The route is not exclusively nodal, because T cells can traffic bidirectionally and dependence on the original draining node can diminish after dissemination (22, 23). Intratumoral antigen-presenting-cell niches provide an additional renewable site, so clone sharing without temporal data does not establish export direction (7). Matched node, blood, and tumor TCR sampling should determine whether nodal expansion precedes blood egress and tumor seeding in the disease phase under study.

Host-to-tumor/node feedback is best illustrated by a bounded microbiota-to-dendritic-cell mechanism. Oral *Lactobacillus rhamnosus* GG activated cGAS–STING-dependent type I interferon in dendritic cells and improved cross-priming and anti-PD-1 activity in mouse colorectal and melanoma models (74). Human microbiome associations, however, are incompletely portable across cohorts and do not show that this pathway operates in every patient (38, 39). They also do not connect the exposure directly to maintenance of a tumor-reactive nodal TCF1-positive reservoir. A prospective test should specify microbial exposure, dendritic-cell interferon and cross-presentation readouts, tumor CD8+ T-cell change, and an external replication cohort, thereby distinguishing directional host calibration from a cross-sectional microbiome signature.

Therapy-to-circuit feedback can repair one compartment while impairing another. Tumor-directed radiation can induce dendritic-cell cGAS–STING/type I interferon signaling, whereas elective irradiation of draining nodes reduced antigen-specific CD8+ T-cell responses and combination efficacy in mouse models (26, 27). Dose and fractionation can further change cytosolic-DNA sensing, making “radiation exposure” too coarse a mechanistic variable. Randomized human radiation-ICB studies have not established a uniform synergy, which limits translation of these preclinical mechanisms (61, 62). Trials should model tumor and uninvolved-node dose separately and relate each to interferon signaling, nodal progenitor states, circulating tumor-matched clones, nonirradiated-lesion response, and regional control. This is a research measurement strategy, not a basis for altering indicated radiation fields.

## 8 Tumor-context variation in candidate bottleneck priors

Tumor type provides a candidate-bottleneck prior, not a fixed assignment. Stage, metastatic organ, genotype, treatment history, nodal state, and host physiology must update it. Three sentinel contrasts illustrate how the same progression endpoint can arise from inadequate antigenicity, regional supply failure, local exclusion, or host calibration without creating six disconnected inventories.

### 8.1 Sentinel contrast: desmoplastic exclusion versus antigenic responsiveness

Unselected pancreatic ductal adenocarcinoma (PDAC) often presents a composite local-access and myeloid problem, but early C-X-C chemokine receptor type 4 (CXCR4)- or focal adhesion kinase (FAK)-directed combinations provide feasibility and pharmacodynamic data, not comparative efficacy (75, 76). Indiscriminate fibroblast or stromal depletion accelerated aggressive disease in experimental PDAC (51, 52), while microsatellite instability-high/mismatch repair-deficient (MSI-H/dMMR) disease is a rare biomarker-defined exception (77). Selective remodeling therefore requires paired access and myeloid readouts plus evidence of recruitable clones. In melanoma, S1801 shows that perioperative timing can matter, whereas NADINA validates a distinct neoadjuvant, response-adapted regimen without isolating timing (78, 79). Regional priming and local differentiation remain candidate mediators: neither trial isolated TDLN function, and nodal metastasis or acquired JAK/B2M lesions may interrupt supply or recognition (17, 25). Longitudinal sampling is therefore required. The contrast is selective local repair versus coordinated use of an existing substrate, not immutable “cold” and “hot” tumor identities.

### 8.2 Sentinel contrast: perioperative benefit versus genotype-defined resistance

In eligible resectable NSCLC, randomized trials support neoadjuvant or perioperative chemo-ICB, directly establishing benefit for the tested regimens (2, 80). AEGEAN and CheckMate 77T provide additional phase III support for their specific perioperative regimens and enrolled molecular populations (81, 82). These results are compatible with treatment while tumor antigen and regional lymphatic tissue remain present, but they do not prove a nodal mediator. CheckMate 722 provides a direct boundary: adding nivolumab to chemotherapy after epidermal growth factor receptor (EGFR) tyrosine kinase inhibitor progression did not improve the primary endpoint in EGFR-mutant metastatic disease (83). Actionable driver, smoking and mutational context, stage, prior therapy, nodal burden, and regimen must therefore be prespecified before assigning a candidate failure site. Pathologic response and event-free survival can document regimen activity, but neither locates the responsible compartment. Perioperative success in driver-filtered disease cannot be generalized to every NSCLC genotype or setting.

A worked test in resectable driver-negative NSCLC would sample baseline tumor and blood, early blood, the surgical tumor, and available regional nodes. Regional supply predicts nodal progenitor expansion before blood emergence and tumor entry. Nodal and blood expansion without tumor entry redirects inference to local access; response without that sequence rejects nodal mediation. CheckMate 722 shifts the clinical prior after EGFR-targeted therapy but cannot identify which of genotype, stage, treatment history, or regimen caused failure.

### 8.3 Sentinel contrast: organ-specific immune calibration

Hepatocellular carcinoma (HCC) shows how organ physiology and treatment setting can reverse a therapeutic expectation. Atezolizumab plus bevacizumab improved outcomes in advanced unresectable HCC, supporting joint checkpoint and vascular targeting in that setting (1). In contrast, the updated IMbrave050 analysis did not sustain the initial recurrence-free-survival advantage after resection or ablation (84). This discordance makes treatment setting a substantive clinical modifier and motivates, but does not establish, a biological explanation. Nonalcoholic steatohepatitis (NASH)-associated immune dysfunction has experimental and retrospective support, but it is not a prospective exclusion rule (85). Etiology, liver function, portal-hypertension and bleeding risk, vascular state, stage, and treatment intent should thus be measured directly. An HCC label alone is insufficient.

Microsatellite-stable (MSS) colorectal cancer and prostate cancer complete the six-context comparison in **Table 2**, avoiding additional mini-reviews. For MSS colorectal cancer, metastatic organ and microsatellite instability/mismatch repair (MSI/MMR) status qualify early combination signals, whereas for unselected metastatic castration-resistant prostate cancer, negative phase III evidence coexists with a tissue-agnostic MSI-H/dMMR exception whose prostate-specific directness is limited (77, 86, 87). Neither context supports unselected combination escalation from mechanism alone. **Table 2** therefore frames each context as a testable intervention or trial-enrichment hypothesis, with evidence directness, candidate measurements, and the principal limitation displayed alongside it.

**Table 2:** Context-informed candidate bottleneck priors and testable trial hypotheses across six tumor types.

Tumor context	Context-informed candidate bottleneck prior (not a validated ranking)	Modifiers that can change the prior	Candidate biomarkers	Testable intervention or trial-enrichment hypothesis	Evidence level and directness	Principal limitation
Pancreatic ductal adenocarcinoma (PDAC)	• Local stromal/vascular and myeloid exclusion is a plausible prior in unselected MSS disease • Weak productive priming may coexist	• MSI-H/dMMR exception (77) • Resectable MRD versus refractory metastatic disease • CAF state, myeloid burden, vascular access, prior chemotherapy, KRAS-antigen detectability	• MSI/MMR • Spatial CD8 access • CAF/TGF-β/ECM state • CXCL12–CXCR4 and FAK pharmacodynamics • Myeloid functional state	Enrich a prospective trial for a measured exclusion/myeloid phenotype; test selective CXCR4/FAK or vascular/myeloid remodeling with an antigen/clone-supply measure and paired access pharmacodynamics. Do not use blanket stromal depletion.	• B, direct early feasibility/pharmacodynamics for CXCR4/FAK combinations (75, 76) • D, direct counterevidence to indiscriminate stromal depletion (51, 52)	• Small, selected, often uncontrolled studies • Stroma has restraining and promoting functions • No comparative evidence ranks compartments; the prior requires prospective testing
Melanoma	• In resectable macroscopic stage III disease, treatment timing is clinically consequential; regional priming/local differentiation remain candidate mediators • Acquired antigen-presentation or IFN lesions may become limiting after response	• Stage and burden • Involved versus uninvolved TDLNs • Prior anti-PD-1 • BRAF status and T-cell-inflamed state	• Pathologic response/EFS • TDLN TPEX and DC organization • Matched node–blood–tumor TCR dynamics • HLA-I/B2M/JAK integrity (17) • Spatial intratumoral and invasive-margin CD8	Embed compartment-resolved sampling within evidence-based perioperative regimens; prospectively test whether nodal TPEX expansion and clone egress mediate response and whether acquired JAK/B2M lesions identify local escape.	• A, direct randomized benefit for tested strategies (78, 79) • B–D, translational/preclinical regional evidence; TDLN mediation remains indirect (9, 25)	• Trials did not randomize a TDLN mediator • NADINA also differed in regimen and postoperative strategy, with more grade ≥3 treatment-related toxicity in the neoadjuvant dual-ICB arm • Findings do not generalize automatically to disseminated or previously resistant disease
Non-small-cell lung cancer (NSCLC)	• In eligible resectable driver-negative disease, local execution and treatment-timed priming are plausible co-bottlenecks • Actionable-driver disease can reorder the hierarchy	• EGFR/ALK and other drivers • Smoking/TMB and PD-L1 • Stage/nodal burden • Histology and perioperative regimen	• Actionable-driver panel • PD-L1/TMB with assay context • Pathologic response and ctDNA/MRD • Tumor–blood–node TCR dynamics • Spatial access and nodal immune state	Stratify perioperative trials by driver status and prespecify tumor, blood, and opportunistic nodal pharmacodynamics; test whether clone expansion adds predictive value beyond tumor-only markers. Do not infer nodal mediation from regimen benefit.	• A, direct regimen efficacy in eligible resectable disease (2, 80–82) • A, direct negative context evidence after EGFR-TKI progression (83)	• Neoadjuvant, chemotherapy, and adjuvant contributions may not be separable • No nodal-preservation randomization • Histologic labels obscure molecular subgroups
Microsatellite-stable colorectal cancer (MSS CRC)	• Low productive antigenicity and local immune exclusion are common priors • Metastatic-organ vascular/myeloid effects may dominate in some patients	• Liver versus lung/peritoneal metastasis • MSI/MMR override • RAS/BRAF, primary site, prior antiangiogenic therapy	• MSI/MMR and relevant genomic exception testing • Metastatic organ • TGF-β/CAF and vascular/myeloid state • Spatial CD8 access and circulating clones	Test vascular/myeloid/stromal remodeling plus ICB only in prospectively biomarker- and metastatic-site-enriched cohorts; validate the non-liver signal in a controlled design.	• B, direct early clinical activity with exploratory organ-specific modifiers (86, 88) • D, direct TGF-β exclusion mechanism (89)	• Non-liver signals are exploratory • No broad efficacy evidence for unselected MSS disease • Multiple co-bottlenecks may prevent single-pathway rescue
Hepatocellular carcinoma (HCC)	• Liver tolerance, VEGF/vascular biology, and myeloid suppression are candidate co-bottleneck priors • Treatment setting changes the prior	• Viral versus nonviral/NASH etiology • Child–Pugh class, portal hypertension, bleeding risk • Macrovascular invasion • Resectable/adjuvant versus advanced disease	• Etiology and liver-function measures • Vascular/myeloid and inflammatory state • Tumor burden/stage and bleeding-risk variables • Paired tumor–blood immune pharmacodynamics	In advanced disease, interpret atezolizumab–bevacizumab within its tested setting. In research, stratify by etiology/liver function and require early vascular/myeloid pharmacodynamics; do not transfer efficacy automatically to adjuvant care.	• A, direct randomized benefit in advanced HCC (1) • A, direct updated counterevidence for routine adjuvant transfer (84) • C–D, NASH modifier evidence (85)	• Liver dysfunction and treatment safety confound immune readouts • Regimen efficacy or non-efficacy does not identify the causal bottleneck; compartmental dominance has not been tested • Etiology findings are not prospective exclusion rules
Prostate cancer	• In unselected mCRPC, low productive immunogenicity plus local/bone-myeloid suppression is a candidate composite prior	• MSI-H/dMMR and other rare genomic exceptions • Bone-predominant versus measurable soft-tissue disease • Histologic transformation • Prior AR-pathway therapy and taxanes	• MSI/MMR and validated genomic exceptions • Antigen-presentation and T-cell-inflamed state • Bone-marrow/myeloid features • ctDNA and measurable-lesion response	Restrict ICB to validated biomarker-defined indications or trials; test priming/vaccination or local/myeloid combinations in genomically and anatomically enriched cohorts with paired pharmacodynamics.	• A, direct negative phase III evidence in unselected mCRPC (87) • B, low but sometimes durable activity in selected phase II patients (90) • B, tissue-agnostic MSI-H/dMMR evidence; prostate-specific directness limited (77)	• Regimen non-efficacy does not identify the causal bottleneck; compartmental dominance has not been tested • Bone lesions are difficult to sample and assess • Responsive biomarker-defined exceptions are rare

Tumor type supplies a prior, not a fixed compartment assignment. Stage, metastatic organ, genotype, prior treatment, spatial immune state, and host factors must update that prior. Every proposed intervention is a trial hypothesis unless already supported for the exact clinical setting; none is a recommendation to depart from standard care. ALK, anaplastic lymphoma kinase; AR, androgen receptor; B2M, β2-microglobulin; CAF, cancer-associated fibroblast; CD8, cluster of differentiation 8; CRC, colorectal cancer; ctDNA, circulating tumor DNA; CXCL12, C-X-C motif chemokine ligand 12; CXCR4, C-X-C chemokine receptor type 4; DC, dendritic cell; dMMR, mismatch repair deficient; ECM, extracellular matrix; EFS, event-free survival; EGFR, epidermal growth factor receptor; FAK, focal adhesion kinase; HCC, hepatocellular carcinoma; HLA-I, human leukocyte antigen class I; ICB, immune checkpoint blockade; IFN, interferon; JAK, Janus kinase; mCRPC, metastatic castration-resistant prostate cancer; MMR, mismatch repair; MRD, minimal residual disease; MSI, microsatellite instability; MSI-H, microsatellite instability-high; MSS, microsatellite stable; NASH, nonalcoholic steatohepatitis; NSCLC, non-small-cell lung cancer; PD-1, programmed cell death protein 1; PD-L1, programmed death-ligand 1; PDAC, pancreatic ductal adenocarcinoma; TCR, T-cell receptor; TDLN, tumor-draining lymph node; TGF-β, transforming growth factor-β; TKI, tyrosine kinase inhibitor; TMB, tumor mutational burden; TPEX, progenitor-exhausted T cell; VEGF, vascular endothelial growth factor.

## 9 Clinical and translational evidence for multiscale intervention

Clinical evidence is strongest for individual strategies and weakest for an integrated circuit sequence. **Table 3** separates randomized regimen efficacy, mechanistic inference, early pharmacodynamic feasibility, negative evidence, and registry-only hypotheses; **Supplementary Table 2** provides the broader intervention catalogue. A phase III trial can be direct for efficacy yet indirect for a compartmental mediator, whereas paired tissue can establish a biological transition without showing that it caused benefit. Mechanistic attribution therefore requires aligned design, endpoint, prespecified sampling, and mediation analysis.

**Table 3:** Sentinel clinical and translational studies that delimit multiscale intervention claims.

Evidence category	Study and setting	Intervention/design	NCT and status on 2026-08-15	Result or current evidence state	Circuit interpretation and principal boundary
Direct regimen efficacy; TDLN mechanism indirect (A)	S1801; resectable stage IIIB–IVC melanoma	Randomized phase II; neoadjuvant–adjuvant versus adjuvant-only pembrolizumab	NCT03698019 Active, not recruiting	Two-year EFS 72% versus 49% (78)	Directly supports the tested timing strategy; tumor/lymphatic basin presence is not a randomized mediator
Direct regimen efficacy; TDLN mechanism indirect (A)	NADINA; resectable macroscopic stage III melanoma	Randomized phase III; neoadjuvant ipilimumab+nivolumab with response-driven adjuvant plan versus surgery then nivolumab	NCT04949113 Active, not recruiting	12-month EFS 83.7% versus 57.2%; HR 0.32; major pathologic response 59.0% (79)	Validates the tested strategy, but arms differed in regimen and postoperative treatment; grade ≥3 treatment-related toxicity was more frequent with neoadjuvant dual ICB; TDLN mediation was not tested
Direct regimen efficacy; TDLN mechanism indirect (A)	CheckMate 816; resectable NSCLC	Randomized phase III; neoadjuvant nivolumab+chemotherapy versus chemotherapy	NCT02998528 Completed	EFS and pCR benefit in the primary report; five-year overall-survival benefit in the mature analysis (2, 91)	Direct regimen benefit; no randomized nodal or regional mediator
Direct regimen efficacy, setting-specific (A)	IMbrave150; advanced/unresectable HCC	Randomized phase III atezolizumab+bevacizumab versus sorafenib	NCT03434379 Completed	Improved randomized clinical outcomes in advanced HCC (1)	Supports vascular–checkpoint co-targeting in the tested setting; does not justify adjuvant transfer
Direct negative/counterevidence, setting-specific (A)	IMbrave050 update; high-risk HCC after resection/ablation	Randomized phase III adjuvant atezolizumab+bevacizumab versus surveillance	NCT04102098 Completed	Updated RFS HR 0.90 and OS HR 1.26; benefit–risk no longer supported adjuvant use (84)	Directly constrains cross-setting extrapolation from advanced HCC
Direct negative regimen efficacy (A)	MASTERKEY-265; advanced melanoma	Randomized phase III T-VEC+pembrolizumab versus placebo+pembrolizumab	NCT02263508 Terminated	No significant PFS or OS improvement (92)	Refutes class-level claims that *in situ* vaccination automatically improves anti-PD-1 efficacy
Direct negative regimen efficacy (A)	ILLUMINATE-301; anti-PD-1-refractory melanoma	Randomized phase III intratumoral tilsotolimod+ipilimumab versus ipilimumab	NCT03445533 Terminated for lack of efficacy	ORR 8.8% versus 8.6%; OS HR 0.96 (93)	Early innate activation did not translate into benefit; TLR agonism is platform- and context-dependent
Direct negative context evidence (A)	CheckMate 722; EGFR-mutant metastatic NSCLC after EGFR-TKI progression	Randomized phase III nivolumab+chemotherapy versus chemotherapy	NCT02864251 Completed	Did not meet the PFS endpoint (83)	Prevents extrapolation from driver-negative perioperative NSCLC to EGFR-mutant metastatic disease
Direct negative regimen efficacy (A)	KEYNOTE-921; previously treated mCRPC	Randomized phase III pembrolizumab+docetaxel versus docetaxel	NCT03834506 Completed	No clinically meaningful randomized benefit in unselected mCRPC (87)	Supports biomarker-defined enrichment rather than routine escalation in unselected prostate cancer
Early LN-targeted immunogenicity; clinical efficacy unproven (B)	AMPLIFY-201/ELI-002 2P; MRD-positive KRAS-mutant PDAC/CRC	Phase I amphiphile vaccine designed for LN delivery	NCT04853017 Completed	mKRAS-specific T-cell responses in 21/25; biomarker clearance in 6/25 (94)	Supports LN delivery and immunogenicity; not TDLN-specific, not combined with ICB, and not randomized for efficacy
Early systemic-conditioning intervention (B)	Davar et al.; anti-PD-1-refractory melanoma	Responder-donor FMT+anti-PD-1; small uncontrolled phase II cohort	NCT03341143 Completed	Clinical benefit in 6/15 with immune/microbial changes (40)	Establishes feasibility and perturbation; donor selection and lack of control prevent added-efficacy attribution
Early negative/limiting intervention evidence (B)	MITRIC; ICB-refractory solid tumors	Phase IIa FMT+ICB, 12 reported patients	NCT05286294 Active, not recruiting	No objective responses; 5/12 stable disease (42)	Demonstrates feasibility without convincing activity in a small heterogeneous cohort

The main table is intentionally limited to sentinel positive and negative studies; the broader intervention and registry catalogue appears in **Supplementary Table 2**. No trial has directly tested the complete proposed sequence; therefore, the available evidence is indirect and hypothesis-generating. This conclusion is bounded to the targeted, non-systematic search through 15 August 2026. Completed component trials must not be combined conceptually into a validated multiscale regimen. Ongoing or active-not-recruiting status does not imply efficacy, availability, or suitability for an individual patient. CRC, colorectal cancer; EFS, event-free survival; EGFR, epidermal growth factor receptor; FMT, fecal microbiota transplantation; HCC, hepatocellular carcinoma; HR, hazard ratio; ICB, immune checkpoint blockade; LN, lymph node; mCRPC, metastatic castration-resistant prostate cancer; mKRAS, mutant KRAS; MRD, minimal residual disease; NCT, ClinicalTrials.gov identifier; NSCLC, non-small-cell lung cancer; ORR, objective response rate; OS, overall survival; pCR, pathologic complete response; PD-1, programmed cell death protein 1; PDAC, pancreatic ductal adenocarcinoma; PFS, progression-free survival; RFS, recurrence-free survival; TDLN, tumor-draining lymph node; TKI, tyrosine kinase inhibitor; TLR, Toll-like receptor; T-VEC, talimogene laherparepvec.

Status date for every NCT entry: 2026-08-15. “Direct regimen efficacy” refers only to the tested population, regimen, and endpoint. It does not imply direct proof of TDLN mediation.

### 9.1 Neoadjuvant and perioperative ICB test timing, not a TDLN mediator

Melanoma trials provide the clearest randomized evidence that treatment timing can matter. In S1801, perioperative pembrolizumab improved two-year event-free survival compared with the same planned number of adjuvant-only doses, while NADINA showed superior event-free survival for neoadjuvant ipilimumab plus nivolumab with response-driven postoperative therapy versus surgery followed by adjuvant nivolumab (78, 79). S1801 is the cleaner same-drug timing comparison. NADINA did not isolate timing because the arms also differed in checkpoint regimen and response-adapted postoperative treatment, and grade 3-or-higher treatment-related adverse events were more frequent with neoadjuvant dual ICB (79). These studies directly validate their tested strategies. They do not randomize tumor-draining-node preservation, distinguish the contribution of involved from uninvolved nodes, or establish that nodal priming caused benefit. Paired tumor-node-blood TCR dynamics and node-state analyses are required to test that mediation. The presence of an intact surgical field is insufficient evidence.

Resectable NSCLC provides convergent strategy-level evidence. CheckMate 816 improved event-free survival and pathological complete response with neoadjuvant nivolumab plus chemotherapy, and its mature analysis reported a five-year overall-survival benefit; KEYNOTE-671 improved event-free survival with a perioperative pembrolizumab regimen (2, 80, 91). The trials establish regimen efficacy in their eligible populations, not universal neoadjuvant superiority, a chemotherapy-to-dendritic-cell mechanism, or TDLN dependence. The negative CheckMate 722 result after EGFR tyrosine kinase inhibitor progression in metastatic disease further shows that molecular, stage, prior-treatment, and disease-setting context constrain extrapolation (83). Opportunistic nodal and blood sampling can add mechanistic resolution, but unsafe biopsies should not be required to preserve the framework.

### 9.2 Local and regional repair remain platform dependent

Human evidence for regional repair is mixed. The lymph-node-targeted amphiphile KRAS vaccine ELI-002 induced antigen-specific T-cell responses in most participants in the phase I AMPLIFY-201 study, supporting delivery and immunogenicity but not TDLN-specific targeting or synergy with ICB (94). Conversely, phase III MASTERKEY-265 did not improve progression-free or overall survival when talimogene laherparepvec (T-VEC) was added to pembrolizumab, and ILLUMINATE-301 found no meaningful benefit from adding intratumoral TLR9 agonism to ipilimumab in refractory melanoma (92, 93). These negative trials show that local antigen release or innate activation is not a validated class effect. Future studies must separate delivery, target engagement, antigen-specific clone induction, and clinical outcome.

Local stromal remodeling is at an earlier stage. CXCR4 inhibition in COMBAT and FAK inhibition with pembrolizumab-based therapy in PDAC produced feasibility and paired immune signals, but their uncontrolled or early-phase designs cannot attribute efficacy to remodeling (75, 76). Small randomized registry results and a terminated anti-TGF-β program add development boundaries and offer no class-wide support (95, 96). Randomized comparison and early spatial, vascular, myeloid, and T-cell pharmacodynamics should precede claims that a local barrier has been repaired.

### 9.3 Systemic conditioning and combined sequences

FMT trials demonstrate that systemic conditioning can be delivered with ICB and can alter microbial and immune states. In refractory melanoma, a small single-arm study reported clinical benefit in a subset after responder-donor FMT plus anti-PD-1, while a separate first-line study reported a high response rate without an ICB-only comparator (40, 41). MITRIC reported no objective responses in a small ICB-refractory cohort, underscoring that feasibility and engraftment do not establish added efficacy (42). A randomized phase Ib/IIa FMT trial in metastatic melanoma is also registered (97). Controlled donor comparison, an ICB-only control where feasible, infection surveillance, and prespecified dendritic-cell and T-cell mediation are needed before microbiome conditioning can be assigned a place in a multiscale sequence.

No trial has directly tested the complete proposed sequence; therefore, the available evidence is indirect and hypothesis-generating. This conclusion is bounded to the targeted, non-systematic search conducted through 15 August 2026 and should not be interpreted as proof of universal absence. Existing studies test perioperative timing, local or nodal activation, stromal remodeling, or microbiome conditioning as separate components. A direct test would require a prespecified sequential or factorial design, compartment-specific mediators, safety and futility rules, and clinical endpoints capable of distinguishing successful target engagement from mere treatment accumulation. Until such evidence exists, the framework should organize experiments and interpret discordant pharmacodynamics, not imply a validated combination strategy.

## 10 Biomarker-guided stratification and adaptive trial design

**Figure 2:**
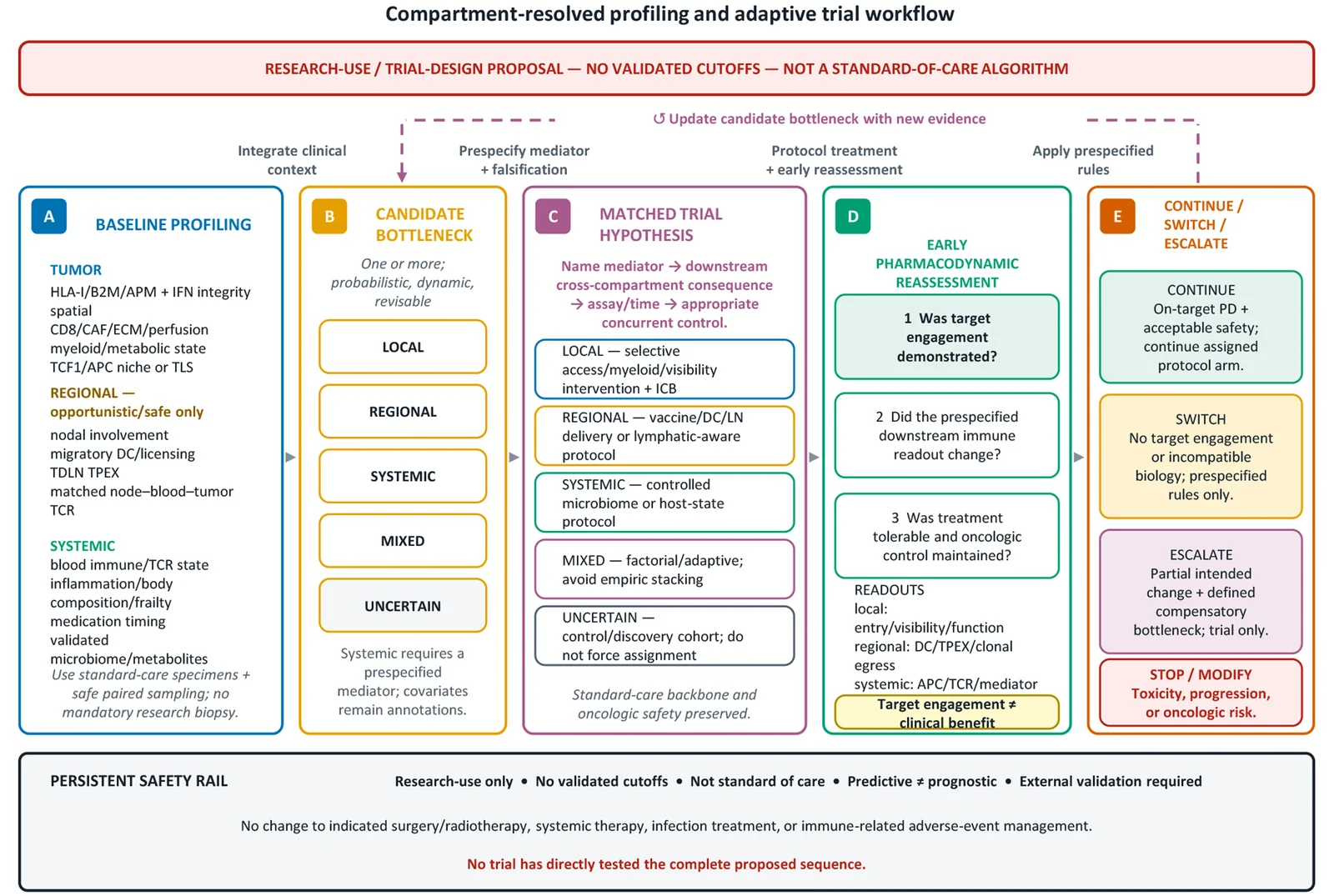
Compartment-resolved profiling and adaptive trial workflow. **(A)** Baseline profiling. **(B)** Candidate bottleneck identification. **(C)** Matched trial hypothesis. **(D)** Early pharmacodynamic reassessment. **(E)** Continue, switch, escalate, or stop/modify according to prespecified rules. The proposed workflow begins with safe, clinically feasible baseline profiling of the tumor, regional lymphatic compartment, and host. The resulting measurements nominate one or more candidate bottlenecks—local, regional, systemic, mixed, or uncertain—without assigning a fixed cancer-type label or numerical cutoff. A matched trial hypothesis then specifies the intervention, intended compartmental mediator, downstream cross-compartment consequence, assay, sampling time, control arm, safety constraints, and clinical endpoint before treatment. Early pharmacodynamic reassessment asks whether the prespecified target and downstream immune state changed; late radiographic response alone cannot establish that the intended bottleneck was repaired. Prespecified decision rules may then continue, switch, or escalate the research arm, with a loop back to the candidate-bottleneck step when target engagement is absent or a compensatory lesion emerges. Human longitudinal studies establish that tumor, blood, and TDLN states and clonotypes can be measured during ICB, but no validated integrated compartment-resolved classifier was identified in this targeted synthesis through 15 August 2026 (5, 21, 25, 65). Randomized perioperative studies validate selected regimens, whereas negative phase III in situ-vaccine/TLR studies demonstrate why mechanistic plausibility and target engagement cannot substitute for clinical benefit (2, 78, 79, 92, 93). This is a research-use framework with no validated thresholds and no authority to alter indicated surgery, radiotherapy, systemic therapy, infection treatment, or immune-related adverse-event management.

The immediate translational use of the framework is to structure measurement and trial hypotheses. It is not to assign routine treatment. **Figure 2** presents the proposed research workflow—baseline profiling, candidate-bottleneck classification, mechanism-matched intervention, and early reassessment—and is explicitly labeled as a trial-design proposal with no validated cutoffs. A compartment-resolved approach must also demonstrate added value over simpler tumor-only markers. More measurements are not informative if they do not improve prediction, identify a mediator, or change a prespecified trial decision.

### 10.1 Baseline compartment-resolved profiling

Baseline profiling should estimate candidate failure sites with assays appropriate to the disease and specimen. Tumor measurements can include HLA-I/B2M and antigen-presentation integrity, interferon-pathway lesions, spatial stromal-versus-parenchymal CD8+ T-cell access, functional myeloid and metabolic states, and TCF1-positive antigen-presenting-cell niches or tertiary lymphoid structures. Regional assessment can use clinically acquired imaging and pathology to distinguish uninvolved from metastatic nodes and, where tissue is available, measure migratory dendritic-cell states, TCF1-positive progenitors, and tumor-related clonotypes. Systemic measures can include blood immune repertoire and phenotype, inflammatory markers, body composition and frailty, medication timing, and analytically validated microbiome or metabolite features. Additional evidence relevant to baseline covariate capture includes diet/probiotic exposure, microbiome-derived metabolites, age-related circulating immune variation, and corticosteroid exposure (98–101). Prospective compartment-resolved sampling could also be embedded in intervention settings such as the registered ELI-002 7P study (102), published responder-donor FMT experience (103), and a randomized phase Ib/IIa FMT trial in metastatic melanoma (97). Cross-cohort microbiome instability and the absence of routine TDLN sampling require missing-data plans and external assay harmonization (25, 38). Candidate assay classes and their limitations are catalogued in **Supplementary Table 1**; none has a validated universal cutoff.

### 10.2 Early pharmacodynamic reassessment

An early on-treatment sample should ask whether the intended compartment changed before a combination is judged only by late imaging. Circulating reinvigoration relative to tumor burden, clonal replacement, peripheral expansion, and nodal progenitor-dendritic-cell dynamics are measurable in human studies, but remain unvalidated candidate mediators (5, 21, 25, 65). Neoadjuvant studies are particularly suited to paired blood, tumor, and opportunistic node sampling because definitive tissue is acquired during planned care. The design should not mandate unsafe biopsies. Sampling windows should be chosen around the expected biological response, and assay failure should be distinguished from absence of target engagement. Serial measurements must be linked by a prespecified temporal model, because a post-treatment association cannot by itself distinguish clone supply, redistribution, and consequence of response.

### 10.3 Trial logic

Trials can enrich or stratify patients by a candidate limiting factor, randomize a mechanism-matched intervention against the relevant control, and specify an early rule for continuation, switching, or escalation. Each arm should declare the targeted compartment and the expected mediator change. A biomarker must be tested for treatment interaction to support predictive use. An outcome association alone may be prognostic. External validation, multiplicity control, assay reproducibility, and comparison with a tumor-only baseline model are required before a complex panel can claim utility. Randomization can be embedded within biomarker strata, while factorial designs can test whether repairing two measured lesions adds more than either intervention alone. Adaptive changes should follow prespecified thresholds, preserve interpretable controls, and avoid adding agents whenever one readout remains abnormal. This logic makes pharmacodynamic failure informative and allows the multiscale model to be rejected if mechanism-matched assignment does not improve outcomes.

Evaluation should report discrimination, calibration, and decision utility in an external cohort and quantify the incremental contribution of regional and systemic measurements after established tumor variables are included. A classifier that improves apparent fit but does not change a prespecified treatment decision, or that fails under assay and center variation, would not justify the added sampling burden. Missing nodal tissue should be modeled explicitly rather than converted into a low regional-risk score.

## 11 Limitations and falsifiable predictions

This framework is derived from a targeted narrative synthesis, with no claim to systematic-review coverage, and its evidence is heterogeneous across species, tumor types, stages, assays, and treatment schedules. Search and selection were designed to revise a defined argument, not to estimate pooled effects or guarantee exhaustive coverage. Many directional mechanisms rely on mouse perturbations, whereas human studies more often provide spatial, clonal, or exposure-outcome associations. TDLNs are not routinely sampled, systemic variables are strongly confounded, and intratumoral antigen-presenting-cell niches or tertiary lymphoid structures may provide anatomical redundancy (7, 9). Sampling itself can select operable disease and accessible nodes, while assay heterogeneity can make the same biological state appear discordant across cohorts. Component interventions have also failed despite compelling mechanisms, including randomized radiation, in situ-vaccine, and TLR9-agonist combinations (62, 92, 93). The framework cannot be protected from negative evidence by retrospectively assigning every failure to an unmeasured bottleneck.

Its value can instead be tested by prespecified refutation criteria. First, a compartment-resolved model should improve externally validated response prediction beyond tumor-only variables. Failure to add predictive value would weaken clinical utility. Incremental performance should be judged with calibration and decision utility, not statistical association alone. Second, where TDLN supply is proposed to dominate, expansion and egress of tumor-related nodal progenitors should precede tumor infiltration. Absence of that temporal sequence would refute the mechanism in that context. Third, a controlled lymphatic-aware or nodal-preservation strategy should improve immune pharmacodynamics without excess regional failure. No benefit, or compromised regional control, would argue against prioritization, and oncologic control of involved nodes must take precedence. Fourth, a microbiome intervention should produce the prespecified dendritic-cell or T-cell mediator change that links exposure to response. Efficacy without that change, or the change without efficacy, would challenge the pathway. Finally, mechanism-matched combinations should outperform empiric escalation within biomarker-defined strata. A null interaction despite confirmed target engagement would be especially informative. If they do not, the framework remains descriptive rather than clinically actionable.

## 12 Conclusion

Resistance to immune checkpoint blockade can sometimes reflect failure of a distributed immune circuit that extends beyond tumor-local defects. Local execution, regional education in tumor-draining lymph nodes, and systemic calibration are functionally nested but anatomically redundant, and reciprocal feedback can shift the candidate limiting factor during treatment. Evidence for individual components is substantial: antigen presentation and access govern local killing, draining nodes can maintain and export tumor-reactive progenitors, and host or treatment exposures can recalibrate dendritic-cell and T-cell states. The evidence is not equivalent across compartments, however, and randomized trials validate selected regimens but not the complete hierarchy.

The near-term implication is therefore methodological. Prospective studies should compare compartment-resolved models with tumor-only baselines, measure early target engagement and clonal dynamics, and specify in advance which observation would disconfirm the proposed mechanism. Tumor, node, blood, body-composition, and microbiome measurements should be selected for biological relevance and feasibility, not assembled as an indiscriminate panel. Until validated cutoffs and treatment interactions are established, this framework is appropriate for trial design and causal interrogation, not routine treatment selection. Its utility will depend on whether it predicts when a locally plausible intervention fails because clone supply or host calibration remains limiting, and whether mechanism-matched testing improves outcomes without compromising standard oncologic control.

## Glossary

APC: antigen-presenting cell
APM: antigen-processing machinery
BATF3: basic leucine zipper ATF-like transcription factor 3
B2M: β2-microglobulin
BMI: body mass index
CAF: cancer-associated fibroblast
cDC1: conventional type 1 dendritic cell
CCR7: C-C chemokine receptor type 7
cGAS: cyclic GMP–AMP synthase
CTLA-4: cytotoxic T-lymphocyte-associated protein 4
CXCR4: C-X-C chemokine receptor type 4
dMMR: mismatch repair deficient
ECM: extracellular matrix
EGFR: epidermal growth factor receptor
FAK: focal adhesion kinase
FMT: fecal microbiota transplantation
G-CSF: granulocyte colony-stimulating factor
HCC: hepatocellular carcinoma
HLA: human leukocyte antigen
HLA-I: human leukocyte antigen class I
ICB: immune checkpoint blockade
IDO1: indoleamine 2,3-dioxygenase 1
IFN: interferon
IL: interleukin
IRF3: interferon regulatory factor 3
JAK: Janus kinase
MHC: major histocompatibility complex
MHC-I: major histocompatibility complex class I
MMR: mismatch repair
MSI: microsatellite instability
MSI-H: microsatellite instability-high
MSS: microsatellite stable
mTOR: mechanistic target of rapamycin
NASH: nonalcoholic steatohepatitis
NOS2: inducible nitric oxide synthase
NSCLC: non-small-cell lung cancer
PD-1: programmed cell death protein 1
PD-L1: programmed death-ligand 1
PDAC: pancreatic ductal adenocarcinoma
PI3Kγ: phosphoinositide 3-kinase-γ
STING: stimulator of interferon genes
TCF1: T cell factor 1
TCR: T-cell receptor
TDLN: tumor-draining lymph node
TGF-β: transforming growth factor-β
TH1: T helper 1
TLR: Toll-like receptor
TME: tumor microenvironment
TPEX: progenitor-exhausted T cell
TREX1: three-prime repair exonuclease 1
T-VEC: talimogene laherparepvec

## References

[1] Finn RS Qin S Ikeda M Galle PR Ducreux M Kim TY . Atezolizumab plus bevacizumab in unresectable hepatocellular carcinoma. N Engl J Med. (2020) 382:1894–905. doi: 10.1056/nejmoa1915745 32402160

[2] Forde PM Spicer J Lu S Provencio M Mitsudomi T Awad MM . Neoadjuvant nivolumab plus chemotherapy in resectable lung cancer. N Engl J Med. (2022) 386:1973–85. doi: 10.1056/nejmoa2202170 35403841 PMC9844511

[3] Siddiqui I Schaeuble K Chennupati V Fuertes Marraco SA Calderon-Copete S Pais Ferreira D . Intratumoral Tcf1+PD-1+CD8+ T cells with stem-like properties promote tumor control in response to vaccination and checkpoint blockade immunotherapy. Immunity. (2019) 50:195–211.e10. doi: 10.1016/j.immuni.2018.12.021 30635237

[4] Miller BC Sen DR Al Abosy R Bi K Virkud YV LaFleur MW . Subsets of exhausted CD8+ T cells differentially mediate tumor control and respond to checkpoint blockade. Nat Immunol. (2019) 20:326–36. doi: 10.1038/s41590-019-0312-6 30778252 PMC6673650

[5] Yost KE Satpathy AT Wells DK Qi Y Wang C Kageyama R . Clonal replacement of tumor-specific T cells following PD-1 blockade. Nat Med. (2019) 25:1251–9. doi: 10.1038/s41591-019-0522-3 31359002 PMC6689255

[6] Torrejon DY Galvez M Abril-Rodriguez G Campbell KM Medina E Vega-Crespo A . Antitumor immune responses in B2M-deficient cancers. Cancer Immunol Res. (2023) 11:1642–55. doi: 10.1158/2326-6066.cir-23-0139 37801341 PMC10842455

[7] Jansen CS Prokhnevska N Master VA Sanda MG Carlisle JW Bilen MA . An intra-tumoral niche maintains and differentiates stem-like CD8 T cells. Nature. (2019) 576:465–70. doi: 10.1038/s41586-019-1836-5 31827286 PMC7108171

[8] Cabrita R Lauss M Sanna A Donia M Skaarup Larsen M Mitra S . Tertiary lymphoid structures improve immunotherapy and survival in melanoma. Nature. (2020) 577:561–5. doi: 10.1038/s41586-019-1914-8 31942071

[9] Connolly KA Kuchroo M Venkat A Khatun A Wang J William I . A reservoir of stem-like CD8+ T cells in the tumor-draining lymph node preserves the ongoing antitumor immune response. Sci Immunol. (2021) 6:eabg7836. doi: 10.1126/sciimmunol.abg7836 34597124 PMC8593910

[10] Prokhnevska N Cardenas MA Valanparambil RM Sobierajska E Barwick BG Jansen C . CD8+ T cell activation in cancer comprises an initial activation phase in lymph nodes followed by effector differentiation within the tumor. Immunity. (2023) 56:107–124.e5. doi: 10.1016/j.immuni.2022.12.002 36580918 PMC10266440

[11] Chen DS Mellman I . Oncology meets immunology: the cancer-immunity cycle. Immunity. (2013) 39:1–10. doi: 10.1016/j.immuni.2013.07.012 23890059

[12] Chen DS Mellman I . Elements of cancer immunity and the cancer-immune set point. Nature. (2017) 541:321–30. doi: 10.1038/nature21349 28102259

[13] Blank CU Haanen JB Ribas A Schumacher TN . Cancer immunology. The "cancer immunogram". Science. (2016) 352:658–60. doi: 10.1126/science.aaf2834 27151852

[14] Hildner K Edelson BT Purtha WE Diamond M Matsushita H Kohyama M . Batf3 deficiency reveals a critical role for CD8alpha+ dendritic cells in cytotoxic T cell immunity. Science. (2008) 322:1097–100. doi: 10.1126/science.1164206 19008445 PMC2756611

[15] Sánchez-Paulete AR Cueto FJ Martínez-López M Labiano S Morales-Kastresana A Rodríguez-Ruiz ME . Cancer immunotherapy with immunomodulatory anti-CD137 and anti-PD-1 monoclonal antibodies requires BATF3-dependent dendritic cells. Cancer Discov. (2016) 6:71–9. doi: 10.1158/2159-8290.cd-15-0510 26493961 PMC5036540

[16] Roberts EW Broz ML Binnewies M Headley MB Nelson AE Wolf DM . Critical role for CD103(+)/CD141(+) dendritic cells bearing CCR7 for tumor antigen trafficking and priming of T cell immunity in melanoma. Cancer Cell. (2016) 30:324–36. doi: 10.1016/j.ccell.2016.06.003 27424807 PMC5374862

[17] Zaretsky JM Garcia-Diaz A Shin DS Escuin-Ordinas H Hugo W Hu-Lieskovan S . Mutations associated with acquired resistance to PD-1 blockade in melanoma. N Engl J Med. (2016) 375:819–29. doi: 10.1056/nejmoa1604958 27433843 PMC5007206

[18] Kamphorst AO Wieland A Nasti T Yang S Zhang R Barber DL . Rescue of exhausted CD8 T cells by PD-1-targeted therapies is CD28-dependent. Science. (2017) 355:1423–7. doi: 10.1126/science.aaf0683 28280249 PMC5595217

[19] Diamond MS Kinder M Matsushita H Mashayekhi M Dunn GP Archambault JM . Type I interferon is selectively required by dendritic cells for immune rejection of tumors. J Exp Med. (2011) 208:1989–2003. doi: 10.1084/jem.20101158 21930769 PMC3182061

[20] Garris CS Arlauckas SP Kohler RH Trefny MP Garren S Piot C . Successful anti-PD-1 cancer immunotherapy requires T cell-dendritic cell crosstalk involving the cytokines IFN-γ and IL-12. Immunity. (2018) 49:1148–1161.e7. doi: 10.1016/j.immuni.2018.09.024 30552023 PMC6301092

[21] Wu TD Madireddi S de Almeida PE Banchereau R Chen YJ Chitre AS . Peripheral T cell expansion predicts tumour infiltration and clinical response. Nature. (2020) 579:274–8. doi: 10.1038/s41586-020-2056-8 32103181

[22] Li Z Tuong ZK Dean I Willis C Gaspal F Fiancette R . *In vivo* labeling reveals continuous trafficking of TCF-1+ T cells between tumor and lymphoid tissue. J Exp Med. (2022) 219:e20210749. doi: 10.1084/jem.20210749 35472220 PMC9048291

[23] Zhao X Kassaye B Wangmo D Lou E Subramanian S . Chemotherapy but not the tumor draining lymph nodes determine the immunotherapy response in secondary tumors. iScience. (2020) 23:101056. doi: 10.1016/j.isci.2020.101056 32344378 PMC7186531

[24] Fransen MF Schoonderwoerd M Knopf P Camps MG Hawinkels LJ Kneilling M . Tumor-draining lymph nodes are pivotal in PD-1/PD-L1 checkpoint therapy. JCI Insight. (2018) 3:e124507. doi: 10.1172/jci.insight.124507 30518694 PMC6328025

[25] Rahim MK Okholm TLH Jones KB McCarthy EE Liu CC Yee JL . Dynamic CD8+ T cell responses to cancer immunotherapy in human regional lymph nodes are disrupted in metastatic lymph nodes. Cell. (2023) 186:1127–1143.e18. doi: 10.1016/j.cell.2023.02.021 36931243 PMC10348701

[26] Deng L Liang H Xu M Yang X Burnette B Arina A . STING-dependent cytosolic DNA sensing promotes radiation-induced type I interferon-dependent antitumor immunity in immunogenic tumors. Immunity. (2014) 41:843–52. doi: 10.1016/j.immuni.2014.10.019 25517616 PMC5155593

[27] Marciscano AE Ghasemzadeh A Nirschl TR Theodros D Kochel CM Francica BJ . Elective nodal irradiation attenuates the combinatorial efficacy of stereotactic radiation therapy and immunotherapy. Clin Cancer Res. (2018) 24:5058–71. doi: 10.1158/1078-0432.ccr-17-3427 29898992 PMC6532976

[28] Spitzer MH Carmi Y Reticker-Flynn NE Kwek SS Madhireddy D Martins MM . Systemic immunity is required for effective cancer immunotherapy. Cell. (2017) 168:487–502.e15. doi: 10.1016/j.cell.2016.12.022 28111070 PMC5312823

[29] Salmon H Franciszkiewicz K Damotte D Dieu-Nosjean MC Validire P Trautmann A . Matrix architecture defines the preferential localization and migration of T cells into the stroma of human lung tumors. J Clin Invest. (2012) 122:899–910. doi: 10.1172/jci45817 22293174 PMC3287213

[30] Mariathasan S Turley SJ Nickles D Castiglioni A Yuen K Wang Y . TGFβ attenuates tumour response to PD-L1 blockade by contributing to exclusion of T cells. Nature. (2018) 554:544–8. doi: 10.1038/nature25501 29443960 PMC6028240

[31] Chang CH Qiu J O'Sullivan D Buck MD Noguchi T Curtis JD . Metabolic competition in the tumor microenvironment is a driver of cancer progression. Cell. (2015) 162:1229–41. doi: 10.1016/j.cell.2015.08.016 26321679 PMC4864363

[32] De Henau O Rausch M Winkler D Campesato LF Liu C Cymerman DH . Overcoming resistance to checkpoint blockade therapy by targeting PI3Kγ in myeloid cells. Nature. (2016) 539:443–7. doi: 10.1038/nature20554 27828943 PMC5634331

[33] Tumeh PC Harview CL Yearley JH Shintaku IP Taylor EJ Robert L . PD-1 blockade induces responses by inhibiting adaptive immune resistance. Nature. (2014) 515:568–71. doi: 10.1038/nature13954 25428505 PMC4246418

[34] Long GV Dummer R Hamid O Gajewski TF Caglevic C Dalle S . Epacadostat plus pembrolizumab versus placebo plus pembrolizumab in patients with unresectable or metastatic melanoma (ECHO-301/KEYNOTE-252): a phase 3, randomised, double-blind study. Lancet Oncol. (2019) 20:1083–97. doi: 10.1016/s1470-2045(19)30274-8 31221619

[35] Dammeijer F van Gulijk M Mulder EE Lukkes M Klaase L van den Bosch T . The PD-1/PD-L1-checkpoint restrains T cell immunity in tumor-draining lymph nodes. Cancer Cell. (2020) 38:685–700.e8. doi: 10.1016/j.ccell.2020.09.001 33007259

[36] Allen BM Hiam KJ Burnett CE Venida A DeBarge R Tenvooren I . Systemic dysfunction and plasticity of the immune macroenvironment in cancer models. Nat Med. (2020) 26:1125–34. doi: 10.1038/s41591-020-0892-6 32451499 PMC7384250

[37] Routy B Le Chatelier E Derosa L Duong CPM Alou MT Daillère R . Gut microbiome influences efficacy of PD-1-based immunotherapy against epithelial tumors. Science. (2018) 359:91–7. doi: 10.1126/science.aan3706 29097494

[38] Lee KA Thomas AM Bolte LA Björk JR de Ruijter LK Armanini F . Cross-cohort gut microbiome associations with immune checkpoint inhibitor response in advanced melanoma. Nat Med. (2022) 28:535–44. doi: 10.1038/s41591-022-01695-5 35228751 PMC8938272

[39] McCulloch JA Davar D Rodrigues RR Badger JH Fang JR Cole AM . Intestinal microbiota signatures of clinical response and immune-related adverse events in melanoma patients treated with anti-PD-1. Nat Med. (2022) 28:545–56. doi: 10.1038/s41591-022-01698-2 35228752 PMC10246505

[40] Davar D Dzutsev AK McCulloch JA Rodrigues RR Chauvin JM Morrison RM . Fecal microbiota transplant overcomes resistance to anti-PD-1 therapy in melanoma patients. Science. (2021) 371:595–602. doi: 10.1126/science.abf3363 33542131 PMC8097968

[41] Routy B Lenehan JG Miller JW Jamal R Messaoudene M Daisley BA . Fecal microbiota transplantation plus anti-PD-1 immunotherapy in advanced melanoma: a phase I trial. Nat Med. (2023) 29:2121–32. doi: 10.1038/s41591-023-02453-x 37414899

[42] Ullern A Garborg KK Chauhan SK Holm K Bang C Dunn C . Safety and efficacy of fecal microbiota transplantation in solid cancers resistant to immune checkpoint inhibitors: results of the MITRIC trial. J Immunother Cancer. (2026) 14:e015122. doi: 10.1136/jitc-2026-015122 42476727 PMC13386059

[43] Schuiveling M Ter Maat LS Van Duin IAJ Verheijden RJ Troenokarso MF Moeskops P . Body composition and checkpoint inhibitor treatment outcomes in advanced melanoma: a multicenter cohort study. J Natl Cancer Inst. (2025) 117:1245–52. doi: 10.1093/jnci/djaf039 39980388 PMC12145918

[44] Zhivaki D Kennedy SN Park J Boriello F Devant P Cao A . Correction of age-associated defects in dendritic cells enables CD4+ T cells to eradicate tumors. Cell. (2024) 187:3888–3903.e18. doi: 10.1016/j.cell.2024.05.026 38870946 PMC11283364

[45] Salih Z Banyard A Tweedy J Galvani E Middlehurst P Mills S . T cell immune awakening in response to immunotherapy is age-dependent. Eur J Cancer. (2022) 162:11–21. doi: 10.1016/j.ejca.2021.11.015 34952479 PMC8829752

[46] Pinato DJ Howlett S Ottaviani D Urus H Patel A Mineo T . Association of prior antibiotic treatment with survival and response to immune checkpoint inhibitor therapy in patients with cancer. JAMA Oncol. (2019) 5:1774–8. doi: 10.1001/jamaoncol.2019.2785 31513236 PMC6743060

[47] Arbour KC Mezquita L Long N Rizvi H Auclin E Ni A . Impact of baseline steroids on efficacy of programmed cell death-1 and programmed death-ligand 1 blockade in patients with non-small-cell lung cancer. J Clin Oncol. (2018) 36:2872–8. doi: 10.1200/jco.2018.79.0006 30125216

[48] Ricciuti B Dahlberg SE Adeni A Sholl LM Nishino M Awad MM . Immune checkpoint inhibitor outcomes for patients with non-small-cell lung cancer receiving baseline corticosteroids for palliative versus nonpalliative indications. J Clin Oncol. (2019) 37:1927–34. doi: 10.1200/jco.19.00189 31206316

[49] Benci JL Xu B Qiu Y Wu TJ Dada H Twyman-Saint Victor C . Tumor interferon signaling regulates a multigenic resistance program to immune checkpoint blockade. Cell. (2016) 167:1540–1554.e12. doi: 10.1016/j.cell.2016.11.022 27912061 PMC5385895

[50] Jacquelot N Yamazaki T Roberti MP Duong CPM Andrews MC Verlingue L . Sustained type I interferon signaling as a mechanism of resistance to PD-1 blockade. Cell Res. (2019) 29:846–61. doi: 10.1038/s41422-019-0224-x 31481761 PMC6796942

[51] Rhim AD Oberstein PE Thomas DH Mirek ET Palermo CF Sastra SA . Stromal elements act to restrain, rather than support, pancreatic ductal adenocarcinoma. Cancer Cell. (2014) 25:735–47. doi: 10.1016/j.ccr.2014.04.021 24856585 PMC4096698

[52] Özdemir BC Pentcheva-Hoang T Carstens JL Zheng X Wu CC Simpson TR . Depletion of carcinoma-associated fibroblasts and fibrosis induces immunosuppression and accelerates pancreas cancer with reduced survival. Cancer Cell. (2014) 25:719–34. doi: 10.1016/j.ccr.2014.04.005 24856586 PMC4180632

[53] Salmon H Idoyaga J Rahman A Leboeuf M Remark R Jordan S . Expansion and activation of CD103(+) dendritic cell progenitors at the tumor site enhances tumor responses to therapeutic PD-L1 and BRAF inhibition. Immunity. (2016) 44:924–38. doi: 10.1016/j.immuni.2016.03.012 27096321 PMC4980762

[54] Lee CYC Kennedy BC Richoz N Dean I Tuong ZK Gaspal F . Tumour-retained activated CCR7+ dendritic cells are heterogeneous and regulate local anti-tumour cytolytic activity. Nat Commun. (2024) 15:682. doi: 10.1038/s41467-024-44787-1 38267413 PMC10808534

[55] Zitti B Duval F Wirapati P Hicham M Xie Y Oh J . Positioning and reversible suppression of CCR7+ dendritic cells in perivascular tumor niches shape cancer immunity. Immunity. (2026) 59:161–176.e12. doi: 10.1016/j.immuni.2025.11.020 41421339 PMC12882814

[56] Pulko V Liu X Krco CJ Harris KJ Frigola X Kwon ED . TLR3-stimulated dendritic cells up-regulate B7-H1 expression and influence the magnitude of CD8 T cell responses to tumor vaccination. J Immunol. (2009) 183:3634–41. doi: 10.4049/jimmunol.0900974 19710456 PMC2789393

[57] Yang H Sun B Ma W Fan L Xu K Jia Y . Multi-scale characterization of tumor-draining lymph nodes in resectable lung cancer treated with neoadjuvant immune checkpoint inhibitors. EBioMedicine. (2022) 84:104265. doi: 10.1016/j.ebiom.2022.104265 36116212 PMC9486045

[58] Woo SR Fuertes MB Corrales L Spranger S Furdyna MJ Leung MY . STING-dependent cytosolic DNA sensing mediates innate immune recognition of immunogenic tumors. Immunity. (2014) 41:830–42. doi: 10.1016/j.immuni.2014.10.017 25517615 PMC4384884

[59] Scarlett UK Cubillos-Ruiz JR Nesbeth YC Martinez DG Engle X Gewirtz AT . In situ stimulation of CD40 and Toll-like receptor 3 transforms ovarian cancer-infiltrating dendritic cells from immunosuppressive to immunostimulatory cells. Cancer Res. (2009) 69:7329–37. doi: 10.1158/0008-5472.can-09-0835 19738057 PMC2754806

[60] Vanpouille-Box C Alard A Aryankalayil MJ Sarfraz Y Diamond JM Schneider RJ . DNA exonuclease Trex1 regulates radiotherapy-induced tumour immunogenicity. Nat Commun. (2017) 8:15618. doi: 10.1038/ncomms15618 28598415 PMC5472757

[61] Theelen WSME Peulen HMU Lalezari F van der Noort V de Vries JF Aerts JGJV . Effect of pembrolizumab after stereotactic body radiotherapy vs pembrolizumab alone on tumor response in patients with advanced non-small cell lung cancer: Results of the PEMBRO-RT phase 2 randomized clinical trial. JAMA Oncol. (2019) 5:1276–82. doi: 10.1001/jamaoncol.2019.1478 31294749 PMC6624814

[62] McBride S Sherman E Tsai CJ Baxi S Aghalar J Eng J . Randomized phase II trial of nivolumab with stereotactic body radiotherapy versus nivolumab alone in metastatic head and neck squamous cell carcinoma. J Clin Oncol. (2021) 39:30–7. doi: 10.1200/jco.20.00290 32822275 PMC8462641

[63] Francis DM Manspeaker MP Schudel A Sestito LF O'Melia MJ Kissick HT . Blockade of immune checkpoints in lymph nodes through locoregional delivery augments cancer immunotherapy. Sci Transl Med. (2020) 12:eaay3575. doi: 10.1126/scitranslmed.aay3575 32998971 PMC8377700

[64] Buchwald ZS Nasti TH Lee J Eberhardt CS Wieland A Im SJ . Tumor-draining lymph node is important for a robust abscopal effect stimulated by radiotherapy. J Immunother Cancer. (2020) 8:e000867. doi: 10.1136/jitc-2020-000867 33028691 PMC7542667

[65] Huang AC Postow MA Orlowski RJ Mick R Bengsch B Manne S . T-cell invigoration to tumour burden ratio associated with anti-PD-1 response. Nature. (2017) 545:60–5. doi: 10.1038/nature22079 28397821 PMC5554367

[66] Coutzac C Jouniaux JM Paci A Schmidt J Mallardo D Seck A . Systemic short chain fatty acids limit antitumor effect of CTLA-4 blockade in hosts with cancer. Nat Commun. (2020) 11:2168. doi: 10.1038/s41467-020-16079-x 32358520 PMC7195489

[67] Nomura M Nagatomo R Doi K Shimizu J Baba K Saito T . Association of short-chain fatty acids in the gut microbiome with clinical response to treatment with nivolumab or pembrolizumab in patients with solid cancer tumors. JAMA Netw Open. (2020) 3:e202895. doi: 10.1001/jamanetworkopen.2020.2895 32297948 PMC7163404

[68] Wang Z Aguilar EG Luna JI Dunai C Khuat LT Le CT . Paradoxical effects of obesity on T cell function during tumor progression and PD-1 checkpoint blockade. Nat Med. (2019) 25:141–51. doi: 10.1038/s41591-018-0221-5 30420753 PMC6324991

[69] McQuade JL Daniel CR Hess KR Mak C Wang DY Rai RR . Association of body-mass index and outcomes in patients with metastatic melanoma treated with targeted therapy, immunotherapy, or chemotherapy: a retrospective, multicohort analysis. Lancet Oncol. (2018) 19:310–22. doi: 10.1016/s1470-2045(18)30078-0 29449192 PMC5840029

[70] Tenuta M Gelibter A Pandozzi C Sirgiovanni G Campolo F Venneri MA . Impact of sarcopenia and inflammation on patients with advanced non-small cell lung cancer (NCSCL) treated with immune checkpoint inhibitors (ICIs): a prospective study. Cancers (Basel). (2021) 13:6355. doi: 10.3390/cancers13246355 34944975 PMC8699333

[71] Gomes F Lorigan P Woolley S Foden P Burns K Yorke J . A prospective cohort study on the safety of checkpoint inhibitors in older cancer patients - the ELDERS study. ESMO Open. (2021) 6:100042. doi: 10.1016/j.esmoop.2020.100042 33516147 PMC7844568

[72] Viaud S Saccheri F Mignot G Yamazaki T Daillère R Hannani D . The intestinal microbiota modulates the anticancer immune effects of cyclophosphamide. Science. (2013) 342:971–6. doi: 10.1126/science.1240537 24264990 PMC4048947

[73] Krall JA Reinhardt F Mercury OA Pattabiraman DR Brooks MW Dougan M . The systemic response to surgery triggers the outgrowth of distant immune-controlled tumors in mouse models of dormancy. Sci Transl Med. (2018) 10:eaan3464. doi: 10.1126/scitranslmed.aan3464 29643230 PMC6364295

[74] Si W Liang H Bugno J Xu Q Ding X Yang K . Lactobacillus rhamnosus GG induces cGAS/STING- dependent type I interferon and improves response to immune checkpoint blockade. Gut. (2022) 71:521–33. doi: 10.1136/gutjnl-2020-323426 33685966 PMC8710942

[75] Bockorny B Macarulla T Semenisty V Borazanci E Feliu J Ponz-Sarvise M . Motixafortide and pembrolizumab combined to nanoliposomal irinotecan, fluorouracil, and folinic acid in metastatic pancreatic cancer: the COMBAT/KEYNOTE-202 trial. Clin Cancer Res. (2021) 27:5020–7. doi: 10.1158/1078-0432.ccr-21-0929 34253578

[76] Wang-Gillam A Lim KH McWilliams R Suresh R Lockhart AC Brown A . Defactinib, pembrolizumab, and gemcitabine in patients with advanced treatment refractory pancreatic cancer: a phase I dose escalation and expansion study. Clin Cancer Res. (2022) 28:5254–62. doi: 10.1158/1078-0432.ccr-22-0308 36228156 PMC9772237

[77] Marabelle A Le DT Ascierto PA Di Giacomo AM De Jesus-Acosta A Delord JP . Efficacy of pembrolizumab in patients with noncolorectal high microsatellite instability/mismatch repair-deficient cancer: results from the phase II KEYNOTE-158 study. J Clin Oncol. (2020) 38:1–10. doi: 10.1200/jco.19.02105 31682550 PMC8184060

[78] Patel SP Othus M Chen Y Wright JG Yost KJ Hyngstrom JR . Neoadjuvant-adjuvant or adjuvant-only pembrolizumab in advanced melanoma. N Engl J Med. (2023) 388:813–23. doi: 10.1056/nejmoa2211437 36856617 PMC10410527

[79] Blank CU Lucas MW Scolyer RA van de Wiel BA Menzies AM Lopez-Yurda M . Neoadjuvant nivolumab and ipilimumab in resectable stage III melanoma. N Engl J Med. (2024) 391:1696–708. doi: 10.1056/nejmoa2402604 38828984

[80] Wakelee H Liberman M Kato T Tsuboi M Lee SH Gao S . Perioperative pembrolizumab for early-stage non-small-cell lung cancer. N Engl J Med. (2023) 389:491–503. doi: 10.1056/nejmoa2302983 37272513 PMC11074923

[81] Heymach JV Harpole D Mitsudomi T Taube JM Galffy G Hochmair M . Perioperative durvalumab for resectable non-small-cell lung cancer. N Engl J Med. (2023) 389:1672–84. doi: 10.1056/nejmoa2304875 37870974

[82] Cascone T Awad MM Spicer JD He J Lu S Sepesi B . Perioperative nivolumab in resectable lung cancer. N Engl J Med. (2024) 390:1756–69. doi: 10.1056/nejmoa2311926 38749033

[83] Mok T Nakagawa K Park K Ohe Y Girard N Kim HR . Nivolumab plus chemotherapy in epidermal growth factor receptor-mutated metastatic non-small-cell lung cancer after disease progression on epidermal growth factor receptor tyrosine kinase inhibitors: final results of CheckMate 722. J Clin Oncol. (2024) 42:1252–64. doi: 10.1200/jco.23.01017 38252907 PMC11095864

[84] Yopp A Chen M Cheng AL Kaseb A Kudo M Lee HC . Updated data from IMbrave050: adjuvant atezolizumab plus bevacizumab for high-risk hepatocellular carcinoma. J Hepatol. (2026) 84:1102–11. doi: 10.1016/j.jhep.2026.01.006 41580093

[85] Pfister D Núñez NG Pinyol R Govaere O Pinter M Szydlowska M . NASH limits anti-tumour surveillance in immunotherapy-treated HCC. Nature. (2021) 592:450–6. doi: 10.1038/s41586-021-03362-0 33762733 PMC8046670

[86] Fakih M Sandhu J Lim D Li X Li S Wang C . Regorafenib, ipilimumab, and nivolumab for patients with microsatellite stable colorectal cancer and disease progression with prior chemotherapy: a phase 1 nonrandomized clinical trial. JAMA Oncol. (2023) 9:627–34. doi: 10.1001/jamaoncol.2022.7845 36892833 PMC9999273

[87] Petrylak DP Ratta R Matsubara N Korbenfeld E Gafanov R Mourey L . Pembrolizumab plus docetaxel versus docetaxel for previously treated metastatic castration-resistant prostate cancer: the randomized, double-blind, phase III KEYNOTE-921 trial. J Clin Oncol. (2025) 43:1638–49. doi: 10.1200/jco-24-01283 40043230 PMC12058370

[88] Fakih M Raghav KPS Chang DZ Larson T Cohn AL Huyck TK . Regorafenib plus nivolumab in patients with mismatch repair-proficient/microsatellite stable metastatic colorectal cancer: a single-arm, open-label, multicentre phase 2 study. EClinicalMedicine. (2023) 58:101917. doi: 10.1016/j.eclinm.2023.101917 37090438 PMC10119887

[89] Tauriello DVF Palomo-Ponce S Stork D Berenguer-Llergo A Badia-Ramentol J Iglesias M . TGFβ drives immune evasion in genetically reconstituted colon cancer metastasis. Nature. (2018) 554:538–43. doi: 10.1038/nature25492 29443964

[90] Antonarakis ES Piulats JM Gross-Goupil M Goh J Ojamaa K Hoimes CJ . Pembrolizumab for treatment-refractory metastatic castration-resistant prostate cancer: multicohort, open-label phase II KEYNOTE-199 study. J Clin Oncol. (2020) 38:395–405. doi: 10.1200/jco.19.01638 31774688 PMC7186583

[91] Forde PM Spicer JD Provencio M Mitsudomi T Awad MM Wang C . Overall survival with neoadjuvant nivolumab plus chemotherapy in lung cancer. N Engl J Med. (2025) 393:741–52. doi: 10.1056/nejmoa2502931 40454642

[92] Chesney JA Ribas A Long GV Kirkwood JM Dummer R Puzanov I . Randomized, double-blind, placebo-controlled, global phase III trial of talimogene laherparepvec combined with pembrolizumab for advanced melanoma. J Clin Oncol. (2023) 41:528–40. doi: 10.1200/jco.22.00343 35998300 PMC9870217

[93] Diab A Ascierto PA Maio M Abdel-Wahab R Negrier S Mortier L . Randomized, open-label, phase III study of tilsotolimod in combination with ipilimumab versus ipilimumab alone in patients with advanced refractory melanoma (ILLUMINATE-301). J Clin Oncol. (2025) 43:1800–9. doi: 10.1200/jco.24.00727 40048691

[94] Pant S Wainberg ZA Weekes CD Furqan M Kasi PM Devoe CE . Lymph-node-targeted, mKRAS-specific amphiphile vaccine in pancreatic and colorectal cancer: the phase 1 AMPLIFY-201 trial. Nat Med. (2024) 30:531–42. doi: 10.1038/s41591-023-02760-3 38195752 PMC10878978

[95] ClinicalTrials.gov . A randomized phase II study of pembrolizumab with or without defactinib, a focal adhesion kinase inhibitor following chemotherapy as a neoadjuvant and adjuvant treatment for resectable pancreatic ductal adenocarcinoma (PDAC). Available online at: https://clinicaltrials.gov/study/NCT03727880 (Accessed August 15, 2026).

[96] ClinicalTrials.gov . A phase II, open label, randomized, parallel arm study of NIS793 (with and without spartalizumab) in combination with SOC chemotherapy gemcitabine/nab-paclitaxel, and gemcitabine/nab-paclitaxel alone in first-line metastatic pancreatic ductal adenocarcinoma (mPDAC). Available online at: https://clinicaltrials.gov/study/NCT04390763 (Accessed August 15, 2026).

[97] ClinicalTrials.gov . Conversion of unresponsiveness to immunotherapy by fecal microbiota transplantation in patients with metastatic melanoma: a randomized phase Ib/IIa trial. Available online at: https://clinicaltrials.gov/study/NCT05251389 (Accessed August 15, 2026).

[98] Spencer CN McQuade JL Gopalakrishnan V McCulloch JA Vetizou M Cogdill AP . Dietary fiber and probiotics influence the gut microbiome and melanoma immunotherapy response. Science. (2021) 374:1632–40. doi: 10.1126/science.aaz7015 34941392 PMC8970537

[99] Mager LF Burkhard R Pett N Cooke NCA Brown K Ramay H . Microbiome-derived inosine modulates response to checkpoint inhibitor immunotherapy. Science. (2020) 369:1481–9. doi: 10.1126/science.abc3421 32792462

[100] Kao C Charmsaz S Tsai HL Aziz K Shu DH Munjal K . Age-related divergence of circulating immune responses in patients with solid tumors treated with immune checkpoint inhibitors. Nat Commun. (2025) 16:3531. doi: 10.1038/s41467-025-58512-z 40258833 PMC12012091

[101] Verheijden RJ de Groot JS Fabriek BO Hew MN May AM Suijkerbuijk KPM . Corticosteroids for immune-related adverse events and checkpoint inhibitor efficacy: analysis of six clinical trials. J Clin Oncol. (2024) 42:3713–24. doi: 10.1200/jco.24.00191 39110922

[102] ClinicalTrials.gov . First in human phase 1/2 trial of ELI-002 7P immunotherapy as treatment for subjects with Kirsten Rat Sarcoma (KRAS)/Neuroblastoma RAS viral oncogene homolog (NRAS) mutated pancreatic ductal adenocarcinoma (PDAC) and other solid tumors. Available online at: https://clinicaltrials.gov/study/NCT05726864 (Accessed August 15, 2026).

[103] Baruch EN Youngster I Ben-Betzalel G Ortenberg R Lahat A Katz L . Fecal microbiota transplant promotes response in immunotherapy-refractory melanoma patients. Science. (2021) 371:602–9. doi: 10.1126/science.abb5920 33303685

