# Supplementary material for: Beyond the tumor microenvironment: a hierarchical immune-circuit model of immune checkpoint blockade resistance
Source: Front Oncol. 2026 Sep 7;16:1919294. doi: 10.3389/fonc.2026.1919294 (PMC13591093; doi:10.3389/fonc.2026.1919294)
Supplement: Supplementary file 1 [file Table1.docx]

Supplementary Material

# Beyond the Tumor Microenvironment: A Hierarchical Immune-Circuit Model of Immune Checkpoint Blockade Resistance

Xue Zhao^¹^, Han Wang^²^, Yanan Liu^²^, Lanqing Cao^²*^

Citation numbers correspond to the reference list in the main manuscript.

## Supplementary Table S1. Candidate compartment-resolved biomarker catalogue

| **Compartment** | **Candidate readout** | **Intended biological question and timing** | **Evidence grade/directness** | **Required boundary for use** |
| --- | --- | --- | --- | --- |
| Local | HLA-I/B2M/APM integrity; JAK1/JAK2 and cell-specific IFN-response state | Is tumor recognition or IFN responsiveness lost?  Baseline and acquired-resistance biopsy | **C**, paired human resistance (17) | B2M loss is context-dependent; add CD4/NK and surface-function assessment (6) |
| Local | Acute versus sustained interferon-stimulated programs | Is IFN licensing present, or has chronic signaling induced counter-regulation?  Serial early/on-treatment sampling | **C–D**, human concordance plus causal mouse studies (49–50) | Bulk ISG abundance cannot identify cell source, duration, or causal direction |
| Local | Stromal-versus-parenchymal/tumor-islet CD8 localization; ECM alignment/density | Is a spatial access bottleneck present?  Baseline and early reassessment | **C**, direct human spatial/migration evidence (29–30) | Invasive-margin CD8 alone is not exclusion and may predict response (33) |
| Local | CAF/TGF-β program, perfusion, endothelial adhesion, and chemokine compatibility | Which structural or vascular feature plausibly blocks entry?  Baseline and mechanism-matched reassessment | **C–D**, human association plus mouse perturbation (30) | Broad stromal/TGF-β targeting is not supported by one signature; stroma may restrain disease |
| Local | Tumor/T-cell glucose uptake; local glucose; T-cell mTOR/glycolysis and IFN-γ | Is local nutrient competition impairing effector function?  Paired pre/post intervention | **D**, direct mouse mechanism (31) | Do not infer systemic glucose manipulation or human efficacy from the model |
| Local | Myeloid PI3Kγ target engagement; macrophage/MDSC functional state | Is suppressive myeloid signaling reversed?  Early pharmacodynamic biopsy/blood pair | **D**, direct genetic/pharmacologic mouse perturbation (32) | Cell abundance alone is inadequate; no validated human efficacy classifier |
| Local | Kynurenine/tryptophan and intratumoral IDO1-pathway target engagement | Is the intended metabolic target inhibited in the tumor?  Early pharmacodynamic sampling | **A** negative regimen evidence; local target engagement indirect (34) | A circulating ratio is not proof of intratumoral target inhibition; ECHO-301 refutes the tested regimen, not all pathway biology |
| Local | TCF1-positive progenitor-to-terminal-state ratio; APC-niche/TLS organization | Does a renewable local ICB-responsive pool exist?  Baseline and early on-treatment | **C–D**, human spatial plus mouse functional evidence (3–4, 7–8) | Phenotype alone does not prove origin; compare matched tumor and TDLN clonotypes |
| Regional | Migratory cDC1 abundance/location, CCR7 trajectory, and shared tumor–node antigen | Is antigen captured and transported productively?  Baseline/neoadjuvant paired sampling | **D**, direct mouse migration perturbation (16, 53) | CCR7 expression alone does not prove completed migration or productive presentation in humans |
| Regional | DC CD80/CD86, type I IFN program, IL-12 cellular source, and DC–T-cell contact | Is antigen display coupled to licensing/costimulation?  Early on-treatment | **D**, direct mouse perturbation (18–20) | Cytokine abundance without cell source/spatial contact is not mechanistic confirmation |
| Regional | TDLN TPEX state and matched node–blood–tumor TCR clonotypes | Does nodal progenitor expansion precede systemic egress and tumor entry?  Serial neoadjuvant sampling | **B–D**, human translational observation plus mouse lineage evidence (9–10, 25) | Clonal overlap does not by itself prove direction; temporal ordering is required |
| Regional | Nodal metastasis, PD-1/PD-L1 cellular contacts, DC/TPEX spatial organization, and suppressive niches | Is the sampled node productive, conditioned, or overtly corrupted?  Baseline/pathology | **B–C**, direct human compartment observation (25, 35, 57) | Anatomical preservation does not guarantee function; involved-node control takes precedence |
| Regional | Circulating tumor-related clonotypes and egress kinetics | Is regional/peripheral clone supply reaching the tumor?  Early serial blood plus tissue | **C–D**, longitudinal human observation plus mouse trafficking perturbation (5, 21–22, 24) | Blood expansion may arise from multiple lymphoid or intratumoral sites; source cannot be assigned from blood alone |
| Regional/therapy | Tumor and uninvolved-node radiation dose, fractionation, cytosolic-DNA/type I IFN readouts | Did radiotherapy activate tumor sensing while preserving regional priming?  During/early after radiotherapy | **D**, direct mouse pathway/field perturbation (26–27, 60) | No validated nodal-dose cutoff for ICB benefit; oncologic target coverage and safety govern treatment |
| Systemic | Blood immune phenotype, proliferative reinvigoration relative to tumor burden, and TCR dynamics | Is a coordinated peripheral response emerging?  Baseline and first treatment cycles | **C**, direct human pharmacodynamic observation (5, 65) | Prognostic and predictive roles are not interchangeable; external validation is required |
| Systemic | APC responsiveness, inflammatory mediators, and myeloid output | Is the host imposing a suppressive activation threshold?  Baseline and early on-treatment | **D** with supportive human observation (28, 36) | Mechanisms vary by model/tissue; a blood signature may not identify the causal organ or mediator |
| Systemic | Standardized community-level microbiome features with diet, geography, antibiotics, and batch metadata | Is a reproducible microbial state associated with response?  Baseline and longitudinal intervention sampling | **C**, multisite human association (38–39, 97) | No universal responder taxon; preanalytics and cohort portability are major limitations |
| Systemic | Microbial/circulating metabolites with receptor/pathway target readout | Is a defined metabolite–immune-cell pathway engaged?  Baseline and pharmacodynamic sampling | **C–D**, human association plus causal mouse mechanism (66–67, 98) | Opposing SCFA associations and context-dependent signaling preclude a generic “beneficial metabolite” score |
| Systemic | Visceral adiposity, muscle quantity/quality, weight trajectory, and cachexia/inflammation | Does body composition reflect modifiable host state or advanced disease burden?  Baseline and longitudinal | **C**, observational human evidence; selected mechanisms **D** (43, 68, 70) | BMI is an imprecise proxy; reverse causation, sex, burden, and performance status must be modeled |
| Systemic | Naive-cell supply, DC migratory function, immune repertoire, biological-age measures, and frailty | Does immune age or reserve modify response or safety?  Baseline and early on-treatment | **C–D**, human phenotyping/frailty plus causal mouse evidence (44–45, 71, 99) | Chronological age is not a resistance class; efficacy and toxicity/reserve endpoints must remain separate |
| Systemic/therapy | Antibiotic/corticosteroid indication, dose, timing, infection, palliative burden, and irAE management | Is treatment exposure a confounder or a plausible circuit perturbation?  Time-varying capture | **C**, observational exposure–outcome evidence (46–48, 100) | Do not withhold medically indicated treatment; confounding by indication and immortal-time bias require explicit analysis |

**Supplementary Table S1 note.** These are candidate diagnostic or pharmacodynamic variables, not validated cutoffs or an integrated clinical classifier. Any predictive model must prespecify assay, timing, treatment, tumor context, missing-data handling, and external validation. Abbreviations: APC, antigen-presenting cell; APM, antigen-processing machinery; B2M, β2-microglobulin; BMI, body mass index; CAF, cancer-associated fibroblast; CD4, cluster of differentiation 4; CD8, cluster of differentiation 8; CD80, cluster of differentiation 80; CD86, cluster of differentiation 86; cDC1, conventional type 1 dendritic cell; CCR7, C-C chemokine receptor type 7; cGAS, cyclic GMP–AMP synthase; DC, dendritic cell; ECM, extracellular matrix; HLA-I, human leukocyte antigen class I; ICB, immune checkpoint blockade; IDO1, indoleamine 2,3-dioxygenase 1; IFN, interferon; IL, interleukin; irAE, immune-related adverse event; ISG, interferon-stimulated gene; JAK, Janus kinase; MDSC, myeloid-derived suppressor cell; mTOR, mechanistic target of rapamycin; NK, natural killer; PD-1, programmed cell death protein 1; PD-L1, programmed death-ligand 1; PI3Kγ, phosphoinositide 3-kinase-γ; SCFA, short-chain fatty acid; STING, stimulator of interferon genes; TCF1, T cell factor 1; TCR, T-cell receptor; TDLN, tumor-draining lymph node; TGF-β, transforming growth factor-β; TLS, tertiary lymphoid structure; TPEX, progenitor-exhausted T cell; TREX1, three-prime repair exonuclease 1.

## Supplementary Table S2. Intervention catalogue and evidence boundaries

| **Candidate intervention or design element** | **Intended compartmental step** | **Current evidence state** | **Required pharmacodynamic test** | **Boundary/counterevidence** |
| --- | --- | --- | --- | --- |
| Perioperative/neoadjuvant ICB in selected melanoma | Expand response while macroscopic antigen and regional circuitry remain present | **A**, direct benefit for S1801/NADINA strategies (78–79) | Pathologic response, TCR dynamics, tumor/TDLN state, EFS | TDLN mechanism is indirect; do not generalize across stages, regimens, or tumors |
| Perioperative chemo-ICB in eligible driver-negative NSCLC | Couple antigen release with checkpoint-responsive clone expansion | **A**, direct regimen benefit (2, 80–82) | pCR/MPR, ctDNA/MRD, clone dynamics, tumor and opportunistic nodal pharmacodynamics | Driver-defined counterevidence; no direct nodal-mediator test (83) |
| Selective CXCR4-axis remodeling in PDAC | Improve local access and alter myeloid trafficking | **B**, early human combination evidence (75) | CXCR4 target engagement, spatial CD8 entry, myeloid state | Uncontrolled efficacy attribution; not a validated PDAC standard |
| FAK inhibition in PDAC | Remodel stromal signaling and improve immune entry | **B**, phase I feasibility/pharmacodynamics (76) | FAK target engagement, CAF/ECM state, paired T-cell entry | Small completed registry trial showed no pCR signal (95) |
| TGF-β/CAF-directed remodeling | Relieve selected fibroblast-mediated exclusion | **C–D**, human association plus mouse reversal (30) | Cell-specific pathway inhibition, tumor-islet CD8 penetration, preserved tissue function | Broad depletion may accelerate PDAC; NIS793 program terminated (51–52, 96) |
| Myeloid PI3Kγ inhibition | Reprogram suppressive macrophage/MDSC function | **D**, direct mouse genetic/pharmacologic evidence (32) | Pathway target engagement, myeloid functional shift, cytotoxic-T-cell restoration | No established human efficacy or universal myeloid biomarker |
| Metabolic competition intervention | Restore local T-cell bioenergetic competence | **D**, glucose-competition mechanism in mice (31) | Compartment-specific substrate availability, T-cell glycolysis/mTOR, IFN-γ | Does not support systemic glucose manipulation; pathway effects vary by tumor and nutrient |
| IDO1 inhibition with anti-PD-1 | Reduce tryptophan–kynurenine suppression | **A**, negative phase III regimen test (34) | Intratumoral target engagement and downstream immune change | Epacadostat+pembrolizumab did not improve PFS/OS; do not equate pathway biology with regimen efficacy |
| Tumor-directed radiotherapy as an in situ immune perturbation | Antigen release and cGAS–STING/type I IFN–dependent cross-priming | **D** mechanism; clinical combinations mixed/negative **B** (26, 60–62) | Dose/fractionation, cytosolic-DNA/IFN pharmacodynamics, nonirradiated-lesion response | Immunogenicity is nonmonotonic; high fractions can induce TREX1; no universal synergy |
| Lymphatic-aware radiation-field design | Preserve uninvolved regional priming while controlling disease | **D**, direct mouse field perturbation (27, 64) | Uninvolved-node dose, nodal TPEX/DC state, systemic clones, regional control | Research question only; no validated human survival benefit or nodal-dose cutoff; involved-node control takes precedence |
| STING/TLR agonism, DC activation, or in situ vaccination | Antigen sensing → type I IFN/DC maturation → cross-presentation/costimulation | **D** mechanism; **A** negative platform-specific phase III trials (20, 58, 92–93) | Innate target engagement, DC licensing, antigen-specific clonal expansion, tumor entry | Early immune activation is not proof of clinical benefit; platform, dose, site, and disease context matter |
| LN-targeted KRAS amphiphile vaccination | Deliver antigen/adjuvant to lymph nodes and generate antigen-specific T cells | **B**, early immunogenicity/MRD signal (94); ongoing efficacy test **E** (101) | LN delivery, KRAS-specific T cells, MRD kinetics, durability | Not proven TDLN-specific; not proof of ICB synergy or survival benefit |
| LN-directed checkpoint delivery | Concentrate checkpoint activity in lymphoid priming sites | **D**, direct preclinical delivery/pharmacodynamic evidence (63) | Nodal exposure, DC/TPEX expansion, systemic clone output, toxicity | Preclinical platform; no validated human regimen or efficacy |
| FMT/microbiome conditioning with ICB | Modify systemic microbial signals and DC/myeloid/T-cell states | **B**, small uncontrolled interventions with positive and negative signals (40–42, 102)  Controlled donor trial ongoing **E** (NCT05251389) | Engraftment, infection safety, metabolite/pathway engagement, prespecified DC/T-cell mediator | Protocolized research only; no randomized added-efficacy proof, universal donor profile, or direct commensal-to-TCF1-reservoir mechanism |
| Body-composition or metabolic-support intervention | Improve systemic reserve and reduce inflammatory/metabolic constraint | Candidate research hypothesis; observational evidence **C** (43, 70) | Longitudinal muscle/adiposity, weight, inflammation, performance, dose intensity | No proof that changing body composition restores ICB response; reverse causation is substantial |
| Antibiotic/corticosteroid exposure-aware trial design | Prevent exposure imbalance and separate treatment effect from indication | Observational evidence **C** (46–48, 100) | Time-varying indication, dose, infection, palliative burden, irAE severity | Not an instruction to withhold medically indicated therapy; causal benefit of avoidance is unproven |
| Complete multiscale sequence | Selective local remodeling → LN/TDLN-targeted activation → systemic conditioning → ICB | **E**, evidence-integrated hypothesis; no direct complete-sequence trial was identified in the targeted search through 15 August 2026 | Prespecified compartmental mediators, order, stopping rules, safety, and clinical endpoints | **No trial has directly tested the complete proposed sequence; therefore, the available evidence is indirect and hypothesis-generating.** This statement is bounded to the targeted, non-systematic search. |

**Supplementary Table S2 note.** Intervention entries are mechanism-matched research hypotheses unless direct clinical benefit is explicitly stated for the exact regimen and setting. Standard oncologic surgery, radiotherapy, systemic therapy, infection treatment, and toxicity management take precedence over unvalidated immune-circuit preservation strategies. Abbreviations: CAF, cancer-associated fibroblast; CD8, cluster of differentiation 8; cGAS, cyclic GMP–AMP synthase; ctDNA, circulating tumor DNA; CXCR4, C-X-C chemokine receptor type 4; DC, dendritic cell; ECM, extracellular matrix; EFS, event-free survival; FAK, focal adhesion kinase; FMT, fecal microbiota transplantation; ICB, immune checkpoint blockade; IDO1, indoleamine 2,3-dioxygenase 1; IFN, interferon; LN, lymph node; MDSC, myeloid-derived suppressor cell; MPR, major pathologic response; MRD, minimal residual disease; mTOR, mechanistic target of rapamycin; NSCLC, non-small-cell lung cancer; OS, overall survival; pCR, pathologic complete response; PD-1, programmed cell death protein 1; PDAC, pancreatic ductal adenocarcinoma; PFS, progression-free survival; PI3Kγ, phosphoinositide 3-kinase-γ; STING, stimulator of interferon genes; TCF1, T cell factor 1; TCR, T-cell receptor; TDLN, tumor-draining lymph node; TGF-β, transforming growth factor-β; TLR, Toll-like receptor; TPEX, progenitor-exhausted T cell; TREX1, three-prime repair exonuclease 1.
